# *Turnip mosaic virus* in oilseed rape activates networks of sRNA-mediated interactions between viral and host genomes

**DOI:** 10.1038/s42003-020-01425-y

**Published:** 2020-11-23

**Authors:** Nicolas Pitzalis, Khalid Amari, Stéfanie Graindorge, David Pflieger, Livia Donaire, Michael Wassenegger, César Llave, Manfred Heinlein

**Affiliations:** 1 Institut de Biologie Moléculaire des Plantes, Centre National de la Recherche Scientifique (IBMP-CNRS), Université de Strasbourg, F-67000 Strasbourg, France; 2 Department of Microbial and Plant Biotechnology, Centro de Investigaciones Biológicas, Consejo Superior de Investigaciones Científicas (CIB-CSIC), Ramiro de Maeztu 9, 28040 Madrid, Spain; 3 RLP Agroscience, AlPlanta–Institute for Plant Research, 67435 Neustadt, Germany; 4 Centre for Organismal Studies, University of Heidelberg, 69120 Heidelberg, Germany; 5 Present Address: Julius Kühn-Institute (JKI), Federal Research Centre for Cultivated Plants, Institute for Biosafety in Plant Biotechnology, Erwin-Baur-Strasse 27, 06484 Quedlinburg, Germany; 6 Present Address: Department of Biology of Stress and Plant Pathology, Centro de Edafología y Biología Aplicada del Segura (CEBAS)-CSIC, 30100 Murcia, Spain

**Keywords:** Bioinformatics, Gene expression analysis, Genomic analysis, RNA, Mechanisms of disease

## Abstract

Virus-induced plant diseases in cultivated plants cause important damages in yield. Although the mechanisms of virus infection are intensely studied at the cell biology level, only little is known about the molecular dialog between the invading virus and the host genome. Here we describe a combinatorial genome-wide approach to identify networks of sRNAs-guided post-transcriptional regulation within local *Turnip mosaic virus* (TuMV) infection sites in *Brassica napus* leaves. We show that the induction of host-encoded, virus-activated small interfering RNAs (vasiRNAs) observed in virus-infected tissues is accompanied by site-specific cleavage events on both viral and host RNAs that recalls the activity of small RNA-induced silencing complexes (RISC). Cleavage events also involve virus-derived siRNA (vsiRNA)–directed cleavage of target host transcripts as well as cleavage of viral RNA by both host vasiRNAs and vsiRNAs. Furthermore, certain coding genes act as virus-activated regulatory hubs to produce vasiRNAs for the targeting of other host genes. The observations draw an advanced model of plant-virus interactions and provide insights into the complex regulatory networking at the plant-virus interface within cells undergoing early stages of infection.

## Abstract (web summary)

Pitzalis et al. use replicative RNAseq, small RNA (sRNA)seq, and parallel analysis of RNA ends (PARE)seq analysis to identify networks of sRNAs-guided post-transcriptional regulation within local Turnip mosaic virus infection sites. This study provides insights into the complex regulatory networking at the plantvirus interface within cells undergoing early stages of infection.

## Introduction

Viral infection in susceptible plant hosts is associated with the altered expression of genes and the activation of pattern-triggered immunity (PTI)^1,2^ and RNA silencing^3^. Both defense responses are triggered upon recognition of double-stranded RNA (dsRNA) produced during viral replication. However, whereas antiviral PTI involves BRI1-ASSOCIATED KINASE 1 (BAK1)^1^ and the recognition of dsRNAs by an unknown receptor^4^, RNA silencing relies on DICER-LIKE (DCL)-mediated cleavage of dsRNA. PTI involves the activation of transcriptional signaling cascades that confer broad-spectrum pathogen resistance^5,6^. In contrast, RNA silencing uses small RNAs (sRNAs), such as small interfering RNAs (siRNAs) and microRNAs (miRNAs), to direct sequence-specific cleavage or translational repression of viral and host RNAs by means of ARGONAUTE (AGO)-containing silencing effector complexes^7^. Besides antiviral defense, RNA silencing plays a central role in plant-virus co-evolution since loss-of-function Arabidopsis mutants defective in RNA silencing genes showed enhanced susceptibility to viral infections^8^. Furthermore, plant viruses encode proteins that actively suppress RNA silencing (viral suppressors of RNA silencing (VSR)). However, the contribution of RNA silencing to the outcome of plant:virus interactions may be more complex than currently known. For example, similar to certain mammalian DNA viruses (Herpesviridae)^9^ and plant-pathogenic fungi^10^, plant viruses may have evolved the ability to exploit their own small RNA repertoire to modulate host gene expression. This may be particularly important during early stages of infection when the virus needs to transform cell functions and suppress stress and defense reactions in newly colonized cells. Following virus entry and replication in the recipient cells, the new viral progeny expands radially from cell to cell conforming the leading front of the spreading infection^11,12^. Cells at the virus front are expected to contain low levels of viral RNA (vRNA) and proteins, which may compromise the ability of VSRs to negatively interfere with RNA silencing-associated regulatory pathways in newly colonized tissues. At the infection front, sRNAs of both viral and host origin may thus gain particular importance by controlling gene and genome expression. Low VSR activity at the front of infection was shown by the inability of tobamoviruses to spread into cells expressing siRNAs targeting the viral genome. Moreover, reducing their VSR activity caused antiviral silencing in cells in the center but not at the spreading leading edge of local infection sites^13,14^. Furthermore, tobacco mosaic virus movement protein, which modifies plasmodesmata (PD) in cells specifically at the virus front^11,15^, enhanced the spread of silencing signal, thus implying a role of small RNA activities in these cells^16^. In addition, exogenous treatment of plants with virus-homologous dsRNA can trigger antiviral silencing and resistance in naive plants, but not in already virus-infected plants^17,18^.

There is growing evidence that plant viruses interfere with sRNA-mediated regulatory pathways to promote changes in gene expression in infected tissues. Certain virus- and viroid-derived siRNAs interact with host-mRNA targets to direct sequence-specific regulation^19–24^, whereas virus-induced changes in miRNA expression reflect changes in the expression of their mRNA targets (e.g., see refs. ^25–27^). Also, secondary siRNAs with potential to guide silencing events are produced from multiple endogenous mRNA transcripts in response to viral infections^28^. However, whereas the cross-talk interaction between plants, viruses and RNA silencing has been primarily studied in systemically infected plants (e.g., see refs. ^24–27,29–39^), very little is known about the regulatory layers that operate between the virus and host cells at the infection front.

Here we uncover a complex network of sRNA-guided regulatory events at the virus infection front by investigating the role of virus- and host-derived sRNAs in guiding RNA target cleavage in the cells of initial sites of infection by *Turnip mosaic virus* (TuMV) within leaves of oilseed rape (*Brassica napus*). Furthermore, we address their potential contribution to viral susceptibility in Drakkar and Tanto cultivars. TuMV is an economically important, aphid-transmitted pathogen in cruciferous plants including oilseed rape and other important *Brassica* species, such as mustard and cabbage. *B. napus* has an allotetraploid genome (2*n* = 4*x* = 38, AACC) and is widely grown for its oil-rich seeds as well as for feed and fuel. A draft genome of the cultivar “Darmor-bzh” was publicly released in 2014^40^. Our results reveal that viral siRNAs as well as siRNAs derived from host coding genes produce a virus-induced sRNA landscape that is used by both the virus and the host to regulate infection. The bidirectional character of these interactions poses a challenge to the current view that RNA silencing is primarily an antiviral defense response. Rather, by allowing mutual regulation, RNA silencing plays a bidirectional role in virus–host compatibility.

## Results

### Virus-induced changes in gene expression

**Fig. 1:**
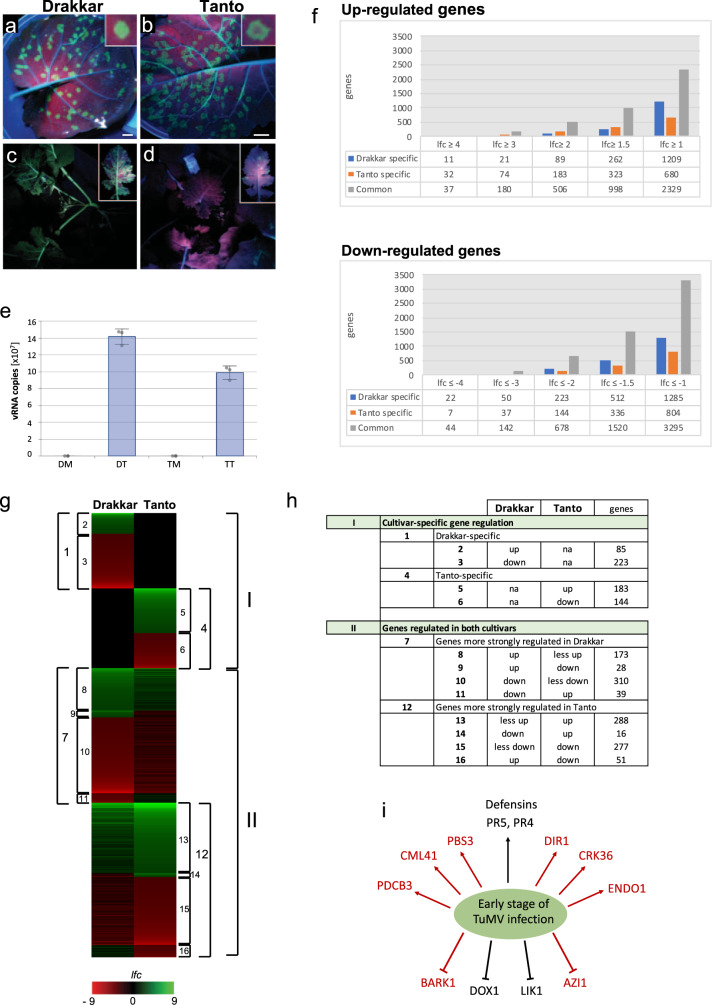
TuMV-GFP infection and virus-induced changes in gene expression in the *B. napus* cultivars Drakkar and Tanto. **a**–**d** Local (6 dpi; **a**, **b**) and systemic (14 dpi; **c**, **d**) TuMV-GFP infection in Drakkar (**a**, **c**) and Tanto (**b**, **d**). Local infection foci in leaves have a disk-shaped appearance in Drakkar (**a**) and a halo-shaped appearance in Tanto (**b**). TuMV exhibits efficient systemic spread in Drakkar (**c***)* but not in Tanto (**d**). **e** Number of TuMV-GFP genome copies within expanded local infection foci at 12 dpi determined by RT-qPCR. The average number of viral genome copies (*n* = 3, with SE) present in total RNA (30 ng) isolated from leaf disks with local infection sites at 12 dpi was calculated by using a RT-qPCR standard curve obtained for samples with known concentrations of in vitro-transcribed TuMV RNA. **f** Number of up- and downregulated genes as determined by RNAseq within local infection foci isolated from infected leaves of Drakkar and Tanto plants at 12 dpi. mRNAs with at least 150 mean reads (treatment and control) in each of three replicate experiments and showing statistically significant changes (*p*-value < 0.05) in expression between control and treatment conditions are considered. The number of differentially expressed genes differs according to the applied log_2_ fold change (*lfc*) threshold. **g** Clustering of virus-induced gene expression patterns at 12 dpi. Only genes showing a *lfc* > 2 are considered. **h** Table legend explaining the specific clusters (cluster 1 to cluster 16) shown in **g**. **i** Summary of up- and downregulated genes. Genes commonly regulated in Drakkar and Tanto are shown in black whereas genes specifically or more strongly regulated in resistant Tanto cultivar are shown in red. Upregulated genes are indicated by arrows. Scale bar in **a** and **b**, 1 cm.

Tanto and Drakkar cultivars of *B. napus* are susceptible for infection by a green fluorescent protein (GFP)-tagged TuMV (TuMV-GFP). Compared to Drakkar, Tanto showed a different distribution pattern of GFP signal within local infection foci (Fig. 1a, b), lower virus accumulation in the inoculated leaves (Fig. 1e), and inhibition of virus systemic movement (Fig. 1c, d), indicating that Tanto is more resistant to TuMV-GFP than Drakkar. Local green fluorescent infection foci in leaves and also leaf samples of similar size of mock-treated plants were excised from three independent biological replicates and used for RNA extraction and transcriptome profiling using RNA sequencing (RNAseq).

Bioinformatic analysis of the transcriptome data revealed a list of virus infection-sensitive transcripts that were either up- or downregulated (*p*-value < 0.05) in both cultivars or in a cultivar-specific manner (Fig. 1f–h). Gene Ontology (GO) annotation enrichment analysis (Supplementary Figs. 1 and  2) suggests that the negative regulation of genes with binding capabilities is partly inhibited in Drakkar with respect to Tanto. In contrast, genes involved in catalytic activities and metabolic processes were significantly repressed in Tanto, but induced in Drakkar. Our data highlight important differences between Tanto and Drakkar in the manner they globally respond to TuMV infection. As summarized in Fig. 1i, upregulated transcripts in both cultivars (see Supplementary Data 1a) included candidates for proteins with homology to Arabidopsis defensin-like proteins and pathogenesis-related protein 4 (PR4) and PR5 (shown in black), whereas highly downregulated transcripts encode proteins with homology to LYSM RLK1-INTERACTING KINASE 1 (LIK1; At3g14840), a leucine-rich repeat receptor-like kinase (LRR-RLK) known to be phosphorylated by CHITIN ELICITOR RECEPTOR KINASE 1^41^, and α-dioxygenase 1 (DOX1)^42^ (shown in black). Figure 1i also summarizes protein-coding genes (shown in red) more induced in Tanto than in Drakkar (see Supplementary Data 1b) or induced in Tanto but not in Drakkar (see Supplementary Data 2b). These genes are predicted to encode the lipid-transfer protein DIR1 required for systemic acquired resistance (SAR) long-distance signaling^43^ and the calcium-binding protein CALMODULIN-LIKE 41 (CML41), which mediates PD closure by callose deposition in response to pathogen attack^44^. Transcripts selectively upregulated in Tanto, but not in Drakkar, encode proteins with similarity to Arabidopsis proteins GH3.12/PBS3, ENDONUCLEASE 1 (ENDO1), PLASMODESMATA CALLOSE-BINDING PROTEIN 3 (PDCB3), and CYSTEINE-RICH RECEPTOR-LIKE PROTEIN KINASE 36 (CRK36). GH3.12/PBS3 is a positive regulator of plant immunity^45^, ENDO1 degrades single-stranded and double-stranded nucleic acids, PDCB3 contributes to callose deposition at PD^46^, and CRK36 positively regulates immunity through interaction with the cytoplasmic kinase BOTRYTIS-INDUCED KINASE 1, a central regulator of PTI that links the activation of pathogen/microbe-associated molecular pattern (PAMP/MAMP) receptor complexes with downstream intracellular signaling^47^. Although further functional studies are needed, these findings suggest that PTI and inhibition of PD conductivity may contribute to the enhanced systemic antiviral resistance in Tanto. On the other hand, Tanto strongly downregulated transcripts of a protein with similarity to the Arabidopsis protein pEARLI1-LIKE LIPID TRANSFER PROTEIN 1 (AZI1) required for signal transmission by the mobile lipid-derived azelaic acid during SAR^48^. Interestingly, AZI1 interacts with PD-LOCALIZED PROTEIN 1, highlighting the regulatory role of PD-localizing proteins in SAR^49^. The mitogen-activated protein kinase kinase kinase (MAPKKK) YODA involved in ERECTA-mediated signaling and immune responses^50^ as well as TMK4/BAK1-ASSOCIATED RECEPTOR-LIKE KINASE 1, a putative membrane LRR-RLK that specifically binds to BAK1^51^, were also strongly downregulated in infected Tanto cells. Further studies may reveal the networking of these specific PAMP- and SA-mediated gene expression responses during virus infection in *B. napus*.

The high degree of identity between homologous and paralogous genes in *B. napus* often hampers the identification of primer pairs to monitor gene-specific changes in transcript levels by reverse-transcription quantitative PCR (RT-qPCR). Nevertheless, we managed to validate virus-induced differential expression of several genes confirming the trends observed by RNAseq (Supplementary Fig. 3). In summary, we found that TuMV infection caused the downregulation of genes for LIK1 and DOX in both cultivars presumably to facilitate viral transmission, whereas the selective induction of  genes for PR5, PR4, DIR1, CML41, PBS3, ENDO1, PDCB3, and CRK36 in Tanto may be linked to specific antiviral responses in this cultivar (Fig. 1i).

### Virus-induced changes in miRNA-mediated target mRNA cleavage

**Fig. 2:**
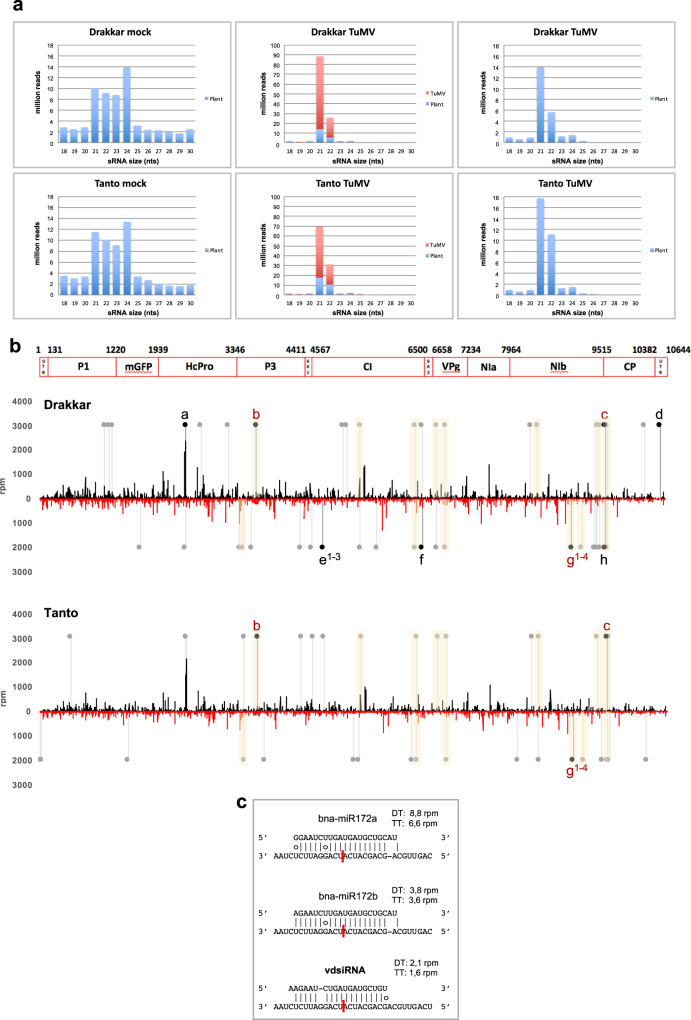
sRNA profile and virus-derived siRNAs (vsiRNAs) predicted to target host genes. **a** Size distribution and normalized abundance of sRNAs in the infected (“Drakkar TuMV” and “Tanto TuMV”) and control (“Drakkar mock” and “Tanto mock”) samples (12 dpi). The abundance of vsiRNAs is shown in red. In the right panels the vsiRNAs counts were removed thus allowing comparison with the left panels to visualize the virus-induced changes in the host sRNA profile. **b** vsiRNA profile and *trans*-acting vsiRNAs predicted to target host genes at 12 dpi in Drakkar and Tanto. vsiRNAs found in Drakkar and Tanto are aligned along the viral genome. vsiRNAs derived from the positive and negative strand of viral dsRNA are shown in black and red color, respectively. Untranslated regions (UTR), proteins encoded by the viral RNA, and nucleotide positions along the viral RNA are indicated at the top of the figure. The vsiRNA profiles are almost identical between Drakkar and Tanto. The positions of vsiRNAs identified by PAREsnip as potentially responsible for specific host-mRNA cleavage are indicated by gray and black dots with lines. Positions of vsiRNAs produced in both cultivars and also associated with mRNA target cleavage in both cultivars are highlighted with light orange color. Positions of vsiRNAs repeatedly associated with the same mRNA target cleavages in several independent degradome libraries (black dots) are indicated by small letter indexes referring to related data shown in Supplementary Data 6 and 7. Red index letters indicate vsiRNAs and corresponding mRNA target cleavage signatures found redundantly in both cultivars. The vsiRNA labeled with “g^1–4^” is predicted to guide the cleavage of conserved sequences in eight different mRNAs encoding four different ethylene-responsive factors (ERFs). **c** The vsiRNA labeled with “g^1–4^” mimics miR172 by causing ERF mRNA cleavage at exactly the same nucleotide position as the miRNA.

The sequenced set of sRNAs showed strong changes in size distribution upon infection (Fig. 2a) with 24 nts representing the major size class in mock-treated plants and 21–22 nts in infected plants. sRNAs (21 nts) identified in virus-infected Drakkar and Tanto plants derived primarily from the virus genome and were named as virus-derived siRNAs (vsiRNAs). Host-derived small RNAs (hsRNAs) of 21 nts showed a conspicuous increase, whereas hsRNAs of 23 and 24 nts were reduced. Alterations in the accumulation levels of sRNAs in host plants leading to an increase in 21/22 nt sRNAs and a decrease in 23/24 nt sRNAs have been previously observed and seem to represent a typical feature of virus-infected plants^52^. vsiRNAs mapped to the TuMV genome along its entire length, on both plus (+) and minus (−) strands (Fig. 2b). The vsiRNA distribution of peaks and valleys was almost identical in Drakkar and Tanto, showing that cultivar-specific differences in viral susceptibility were not due to different antiviral DCL activities in each cultivar.

**Fig. 3:**
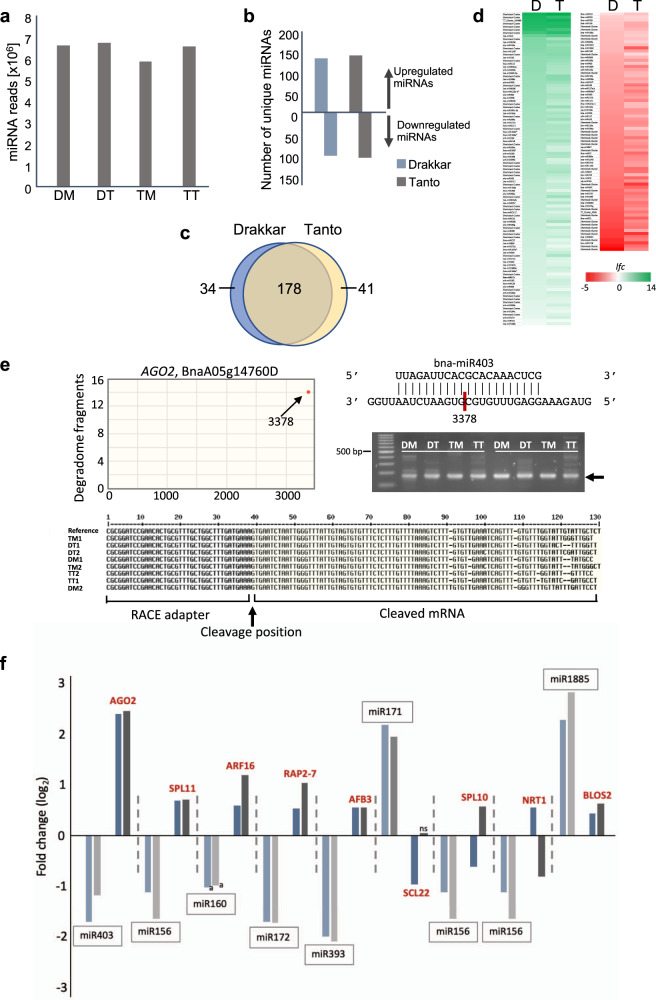
Virus-induced changes in miRNA abundance and activity. **a** Total normalized miRNA reads in the three replicates per treatment. **b** Upregulated and downregulated miRNA species in Drakkar and Tanto. Only miRNAs showing significant changes in abundance (*p* < 0.05) are considered. **c** Cultivar-specific and common miRNAs showing significant changes in abundance (*p* < 0.05). **d** Virus-induced changes in miRNA levels. D, Drakkar; T, Tanto; *lfc*, log_2_ fold change. **e** Site-specific cleavage of *AGO2* transcripts at the predicted miR403-binding site. Target plot for degradome profiling of *AGO2* transcripts showing preferential 5′ signatures at the bna-miR403-binding site at position 3378 (top left). Base-pair alignment between bna-miR403 and *AGO2* transcripts showing the specific cleavage site at position 3378 of the mRNA, and 5′-RACE analysis of two independent biological samples showing DNA fragments of the predicted length (top right). Site-specific cleavage at position 3378 was confirmed by DNA sequencing of the 5′-RACE DNA fragments (bottom). **f** Virus-induced changes in the abundance of both miRNA species in Drakkar (light blue) and Tanto (light gray) and their mRNA targets (Drakkar, dark blue; Tanto, dark gray) found to be cleaved in our degradome analysis. mRNAs are annotated according to their predicted proteins they encode (in red).

In this study, we identified 1047 unique miRNA sequences. The number of sequenced 21 nt miRNA reads was similar between the mock and virus-treated samples (Fig. 3a), although TuMV infection caused strong alterations in the levels of unique miRNAs. Importantly, the collective number of unique miRNAs that were either up- or downregulated (*p* < 0.05) upon infection was similar between the two cultivars (Fig. 3b). Among the 253 miRNAs that were represented in the samples by at least 10 mean normalized reads, 178 individual miRNAs detected in both cultivars (Fig. 3c) showed almost identical changes in their levels upon infection (*p* > 0.05) (Fig. 3d and Supplementary Data 3). Thus, although infection causes strong changes in unique miRNA levels, which presumably impact the amplitude of miRNA-mediated cleavage of their cognate mRNA targets, miRNA levels seem to be unrelated to cultivar-specific susceptibility for the virus.

RNA ligase-mediated (RLM)-5′-rapid amplification of cDNA ends (5′RACE) enabled us to identify sequence-specific cleavage breakpoints for several representative miRNA:mRNA target pairs (Fig. 3e; see also Supplementary Figs. 4–12). Among them, we found that bna-miR1885a, previously reported to target a *B. napus * Toll/interleukin receptor, nucleotide-binding site (TIR-NBS)-RLK resistance gene^53^, guided cleavage at the center of the miR1885a-binding site within a BnaA09g14980D transcript encoding a protein with 70% similarity to the Arabidopsis TARGET OF AVTB OBERATION 1 (TAO1) (Supplementary Fig. 7). Experimental evidence for target cleavage in a transcriptome-wide scale was obtained by PAREseq^54^ using home-made libraries of 5′-degradome tags and PAREsnip for analysis^55^. As opposed to 5′-RACE, degradome-based analyses allow us to repetitively identify and validate cleavage events in a transcriptome-wide scale and to correlate them with evidence for sequence-specific pairing between sRNA/miRNA and the specific targets. As predicted, several well-known miRNA–mRNA target pairs that fulfilled our requirement for expressed target mRNAs (at least 150 mean reads) were found in at least three out of 12 samples used for PARE analysis (Supplementary Data 4). As previously reported, degradome 5′ tags diagnostic of the expected miRNA-guided cleavage were either the most abundant tags matching the transcript or formed a conspicuous peak at the complementary site.

In our analysis, miRNAs and their mRNA targets usually exhibited an inverse correlation in their accumulation profiles in both cultivars (Fig. 3f and Supplementary Data 5). Yet, a few other miRNA–mRNA pairs showed no such a correlation. For example, the strong virus-induced accumulation of miR1885b was accompanied by a subtle increment of its predicted target transcripts in both cultivars. This scenario may illustrate a fine-tuned miRNA-driven regulation that prevents the excessive accumulation of target transcripts that are upregulated during infection, as recently described for several plant immune receptors^2,56,57^. In other instances, negative correlation was seen in only one of the two cultivars, which could be caused by cultivar-specific differences in the expression of the target gene. Among them, we found specific transcripts for SQUAMOSA PROMOTER BINDING PROTEIN LIKE  (SPL) that were targeted by miR156d,e,f in Tanto (Supplementary Data 5). Collectively, our degradome data indicate that miRNA–mRNA pairs previously reported in Arabidopsis and other species were also active in *B. napus* and suggest a functional role of miRNAs in specific gene regulation during infection.

### Host-mRNA cleavage by vsiRNAs

Inspired by the evidence that fungal pathogens employ siRNAs for cross-kingdom silencing to enhance compatible interactions with their plant hosts^10^, we wondered whether TuMV may also use its own repertoire of siRNAs to target host genes at the initial sites of infection and during spreading. Our degradome data revealed sequence-specific cleavage events in 205 host mRNAs and identified 87 vsiRNAs as potentially responsible for cleavage. 5′-degradome tags suggestive of vsiRNA-guided cleavage were detected exclusively in the infected tissues but not in mock-treated controls, which strongly supports functional interactions between vsiRNAs and their targets (Supplementary Data 6). These cleavage-associated siRNAs derived from both minus and plus strands of the virus and mapped at various positions along the viral genome (Fig. 2b). Eight of them (referred to as “a to h”) stood out from the crowd of vsiRNAs, as they were repetitively associated with cleavage events in 17 different transcripts, and were detected in at least two of the six independent degradomes from infected plants (Fig. 2b and Supplementary Data 7). These vsiRNAs, except “g” may have potential to cleave additional transcript targets (Supplementary Data 6). The vsiRNA indexed with “g” (5′-AAGAATCTGATGATGCTGT-3′) shows high sequence similarity to Bna-miR172, which is known to target several AP2-like ethylene response factors (ERFs)^58,59^. Consistently, similar to miR172, this vsiRNA had sequence complementarity with transcripts of eight genes encoding ERFs, RAP2–7, TOE2, and APETALA2 (indexed with “g” in Fig. 2b and Supplementary Data 6), and it is predicted to bind at exactly the same position as miR172 within the target sequence (Fig. 2c; for more details, see Supplementary Figs. 7 and  13). This observation suggests that both miR172 and miR172-like vsiRNAs may cooperate in regulating miR172 targets in infected plants as deduced by degradome data. Our PAREsnip analysis also provided experimental evidence of endonucleolytic cleavage of gene transcripts encoding the pseudokinase ZED1, the cysteine-protease aleurain and the serine/arginine-rich (SR) RS31A, and identified several other vsiRNAs as candidate cleavage guides. The pseudokinase ZED1 is an important mediator of effector-triggered immunity^60^, whereas the cysteine-protease aleurain plays an important role in hypersensitive responses and programmed cell death^61^. SR proteins are essential factors in regulating pre-mRNA constitutive and alternative splicing^62^. They also act in RNA surveillance and promote RNA degradation through the nonsense-mediated RNA decay pathway^63^. In plants, SR proteins are involved in the splicing of RNA encoding plant immune receptors^64^ and play numerous roles in stress tolerance^65^. We observed that mRNA cleavage events directed by vsiRNAs correlate with only moderate, albeit significant, changes in target transcript accumulation (Supplementary Data 6). This finding suggests that the spreading virus exploits siRNAs of viral origin to control, but not necessarily downregulate, the expression of several host target genes.

### Infection induces the production of virus-activated siRNAs from coding genes

**Fig. 4:**
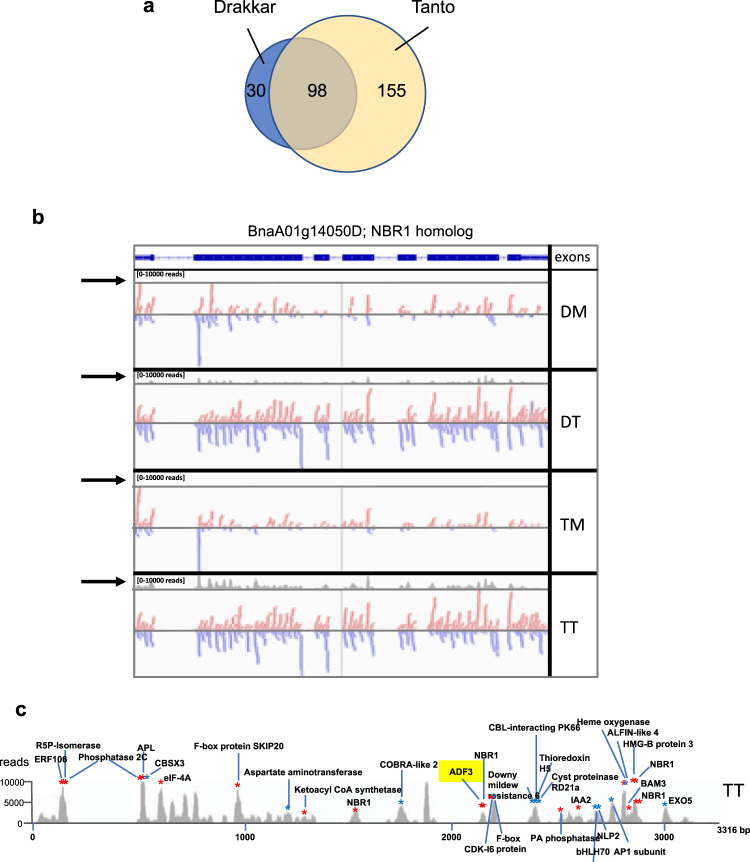
Infection induces the production of host-encoded vasiRNAs with potential to target other host genes. **a** Number of vasiRNA-producing loci in Drakkar and Tanto. **b** vasiRNAs produced by the *NBR1* gene (BnaA01g14050D). Unique vasiRNAs sequenced in DM, DT, TM, and TT samples align along the exonic regions of the gene transcript. Arrows indicate diagrams showing the abundance of the vasiRNAs aligned to the transcript (scale: 0–10000 reads). **c** *Trans*-acting vasiRNAs derived from *NBR1*. vasiRNAs associated with mRNA target cleavage signatures are derived from the peaks of vasiRNA production. The vasiRNA peaks from infected Tanto (TT) samples are shown (peaks in the DT sample are similar, but not as high). Red and blue dots indicate vasiRNAs derived from the positive and negative strands of the *NBR1* double-stranded RNA, respectively. A peak of vasiRNAs that target transcripts from *ADF3* is highlighted in yellow.

Our degradome analysis identified two AGO2-coding transcripts (BnaCnng68320D and BnaA05g14760D) as potential targets of miR403 in mock-treated and TuMV-infected *B. napus* samples (Fig. 3e and Supplementary Data 4 and 5). Interestingly, TuMV infection triggered the production of siRNAs from *AGO2* transcripts in both Drakkar and Tanto plants (Supplementary Fig. 14), which resemble the virus-activated siRNAs (vasiRNAs) described in other plant-virus systems^28^. The production of vasiRNAs was accompanied by a downregulation of miR403 along with increasing levels of *AGO2* transcripts (Fig. 3f and Supplementary Data 5). Although none of the *AGO2*-derived vasiRNAs was associated with sRNA-mRNA target pairs indicated  by PAREsnip, the production of secondary siRNA may provide a feedback mechanism for controlling an excess of *AGO2* transcripts in infected cells as recently reported^28^. We further identified 128 and 253 coding genes predicted to act as vasiRNA-producing loci in Drakkar and Tanto, respectively (Supplementary Data 8). Among them, 98 coding genes act as vasiRNA-producing loci in both cultivars (Fig. 4a and Supplementary Data 9).

The vasiRNA-producing loci include members of two small gene families predicted to encode CHLOROPHYLL A-B BINDING PROTEIN 1 (CABBP1) and a Autophagy Cargo Receptor NBR1 homolog, respectively. The high amounts of secondary siRNAs produced from *NBR1* during infection correlated with high transcript levels as previously shown with *AGO2* (Supplementary Data 10). Importantly, our PAREsnip analysis associated 71 and 89 unique vasiRNAs produced from *CABBP1* and *NBR1* genes (Supplementary Data 11) with cleavage events in 106 and 163 unique vasiRNA - mRNA target pairs, respectively (Supplementary Data 12). Furthermore, degradome sequencing showed evidence for cleavage of vasiRNA targets in infected tissues but not in mock-inoculated controls, suggesting that cleavage events linked to *CABBP1*- and *NBR1*-derived vasiRNAs occur predominately in response to TuMV infection. Interestingly, the targets of *CABBP1*- derived vasiRNAs corresponded to transcripts of *CABBP1* genes, indicating that these vasiRNAs primarily targeted transcripts from homologous genes. In contrast, 5′ cleaved tags derived from mRNA transcripts of several genes, such as mRNA transcripts encoding ACTIN DEPOLYMERIZATION FACTOR (ADF), were suggestive of cleavage guided by vasiRNAs derived from *NBR1*, suggesting that vasiRNAs may interact *in trans* with mRNAs from other loci (Supplementary Data 12 and 13). RNAseq data indicated that the targets of CABBP1-derived vasiRNAs were downregulated in Drakkar, whereas they were induced in Tanto (Supplementary Data 12 and 13). In contrast, the ADF-encoding genes targeted by *NBR1*-derived vasiRNAs were downregulated during infection in both cultivars (Supplementary Data 13). As ADF3 plays an important role in plant defense against aphids^66^, a negative regulation by vasiRNAs may facilitate virus transmission. Interestingly, the *NBR1*-derived vasiRNAs predicted to interact with secondary gene targets mapped exactly to the peaks of vasiRNA production along the *NBR1* transcript (Fig. 4b, c). This particularity suggests that vasiRNAs associated with target cleavage events are stabilized by association with their effector complexes.

Taken together, these observations provide solid experimental evidence that virus infection in oilseed rape triggers the production of secondary vasiRNAs from numerous coding genes, and that many of these vasiRNAs are competent to promote sequence-specific cleavage of target mRNAs encoded by the same gene family (e.g., *CABBP1*) or by other genes (e.g., *NBR1*).

### Identification of host-derived siRNAs targeting vRNA for cleavage

**Fig. 5:**
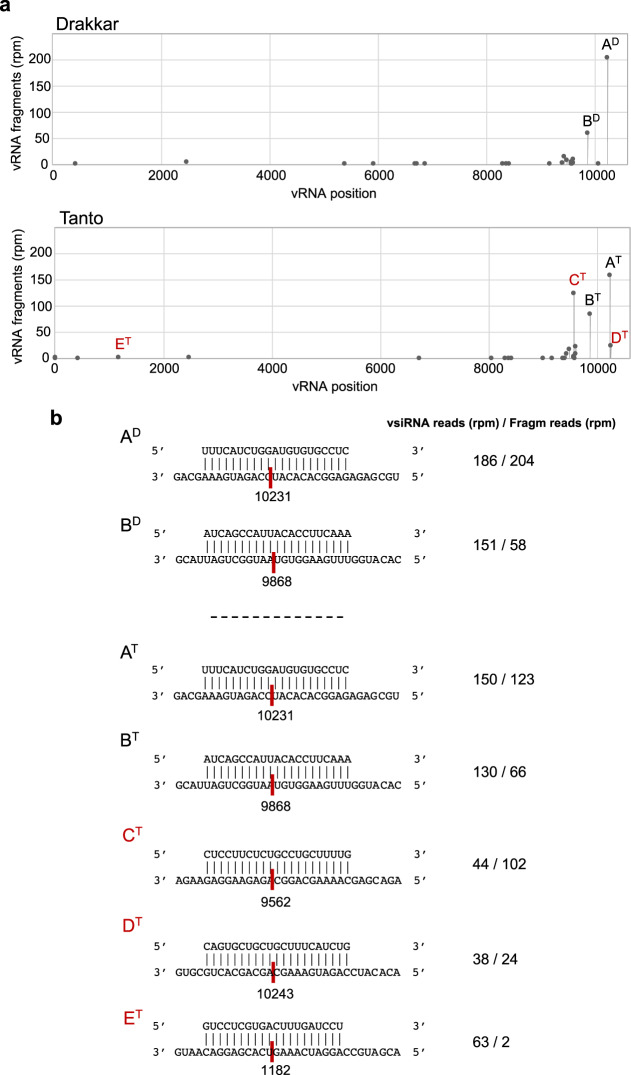
vsiRNA-guided cleavage of viral RNA. **a** Target plots showing 5′-signature abundance along the viral RNA identified through degradome sequencing of local infection sites in Drakkar and Tanto leaves (Top). A to E letters in the target plots denote cleaved positions represented by highly abundant signatures that occurred either in both cultivars (A^D^/A^T^; B^D^/B^T^), with higher frequency in Tanto (C^T^) or even exclusively only in Tanto but not in Drakkar (D^T^ and E^T^). **b** Base-pair alignments between viral RNA and vsiRNA at A to E positions are shown together with normalized abundance (r.p.m.) for the specific vsiRNA and the normalized abundance (r.p.m.) of the corresponding viral RNA cleavage fragment in the degradome library.

We wondered whether the plant may use endogenous host-derived siRNAs (hsiRNAs) to target the vRNA for degradation. To approach this question, we searched our degradome data for virus-specific signatures derived from the (+) and (−) vRNA strands. Several of the virus-specific signatures could be associated with vsiRNA-vRNA target pairs. These revealed 56 unique vsiRNAs derived from the negative strand of the virus as plausible guides for cleavage of vRNA (Supplementary Data 14 and Fig. 5). Among them, 29 target pairs were found in both Drakkar and Tanto. 5′-Degradome signatures were particularly abundant at nucleotide positions 9868 and 10,231 (referred to as A and B) in both cultivars, suggesting that they represent prominent cleavage sites within the viral genome. They were clearly discerned from a background of low abundant signatures that likely represent suboptimal cleavage sites. Three additional sites of preferential cleavage at positions 9562, 10,243, and 1182 (referred to as C, D, and E) were predominantly found in Tanto (C) or exclusively identified in Tanto, but not in Drakkar (D and E). As vsiRNAs are produced at very high levels from all along the viral sequence (Fig. 2), these observations suggest certain selectivity for specific vsiRNAs engaging in vRNA cleavage.

**Fig. 6:**
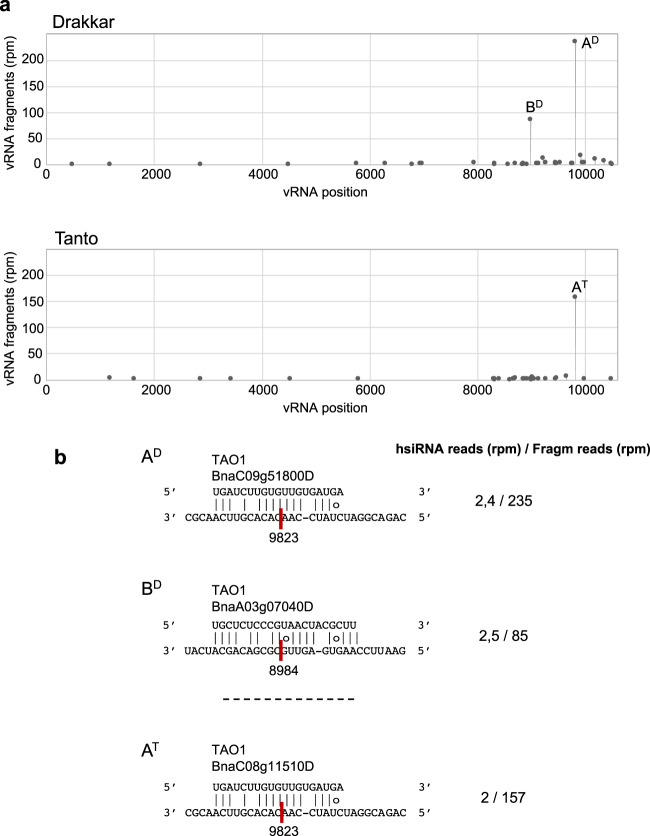
Sequence-specific cleavage of viral RNA at predicted hsiRNA-binding sites. **a** Target plots showing 5′-signature abundance along the viral RNA identified through degradome sequencing of infected Drakkar and Tanto (top). Cleavage sites represented by the most abundant 5′-degradome signatures within the target plots (referred to as A and B) involve siRNAs derived from genes predicted to encode the TIR-NB-LRR disease resistance protein TAO1. **b** Base-pair alignments between viral RNA and hsiRNA at A and B positions (bottom). Viral RNA cleavage sites and viral RNA cleavage positions are shown together with normalized abundance (r.p.m.) for the specific hsiRNA and the normalized abundance (r.p.m.) of the corresponding viral RNA cleavage fragment in the degradome library.

In addition to vsiRNAs, we found 87 unique hsiRNAs with potential to base-pair with specific sites of cleavage along the vRNA (Fig. 6). Forty-five and 64 of them were found in Drakkar and Tanto, respectively, whereas 23 of them were shared between both cultivars (Supplementary Data 15). The identification of identical vsiRNA-vRNA and hsiRNA - vRNA pairs in the two cultivars supports the notion that site-specific RNA cleavage by host-derived siRNAs could be a major point of viral regulation during infection. Several of the hsiRNA-mediated cleavage events involved *trans*-acting vasiRNAs derived from NBR1 genes (Supplementary Data 15). However, the large majority of them were associated with unique hsiRNAs derived from the disease resistance genes *TAO1* and *RPP5*, both encoding TIR-NB-LRR receptor-like proteins (Fig. 6). These and other hsiRNAs involved in viral targeting events arise from genomic loci that normally produce numerous secondary siRNAs in both mock-treated and TuMV-infected plants (Supplementary Fig. 15), suggesting the possibility that these resistance genes pre-immunize the plant by producing antiviral siRNAs prior to infection. Collectively, our analysis revealed that during infection both Drakkar and Tanto produce a large pool of vasiRNAs and hsiRNAs with the potential to assist or compete with vsiRNAs in guiding sequence-specific degradation of vRNA.

## Discussion

**Fig. 7:**
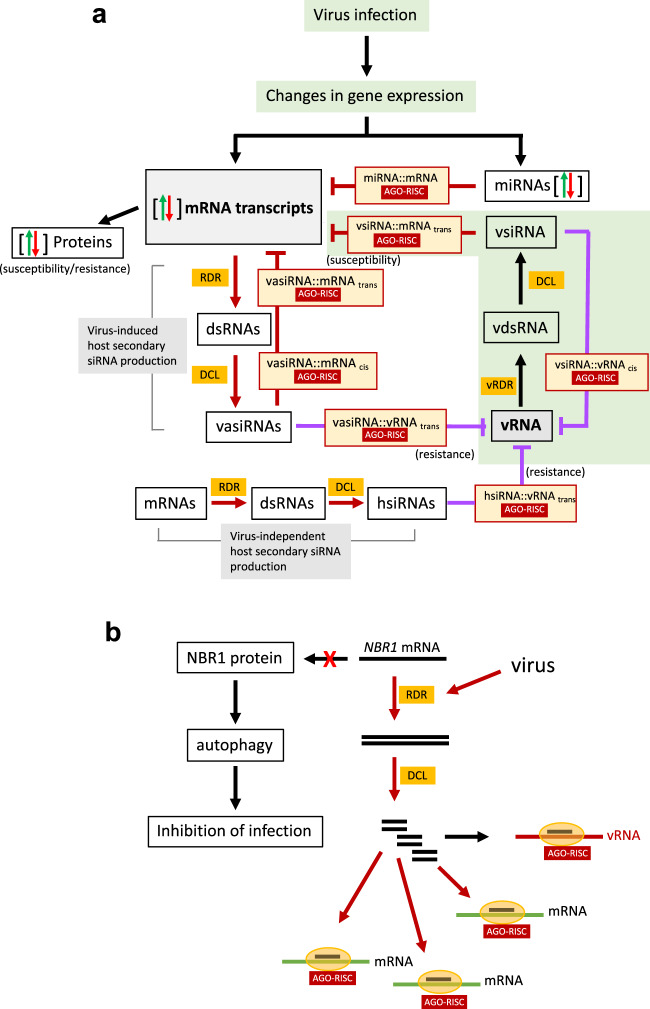
Model of virus-host interactions mediated by sRNAs. **a** Virus infection induces a complex regulatory matrix of bidirectional sRNA-mediated interactions at the plant:virus interface. Virus infection causes changes in the expression of miRNAs and mRNAs that may be critical in the determination of host susceptibility and resistance. Perturbations in miRNA levels may affect the amplitude and efficacy of miRNA-mediated cleavage resulting in changes in the level of their mRNA targets. Virus infection also triggers the biosynthesis of vasiRNAs from host transcripts via the formation of dsRNA intermediates. Recruitment of vasiRNAs into cleavage-effector silencing complexes provides control over a vast range of host transcripts including transcripts of the vasiRNA-producing genes and their homologs (e.g., vasiRNAs produced by *CABBP1*) and transcripts derived from secondary target genes (e.g., vasiRNAs produced by *NBR1*). The replicating virus uses dsRNA replicative intermediates to produce vsiRNAs which are competent to guide sequence-specific cleavage of both viral and host-mRNA targets. Likewise, viral RNA is an accessible target for vsiRNA-, vasiRNAs, and hsiRNA-directed cleavage. hsiRNAs are produced by the host also in the absence of virus and may contribute to basal resistance of the host. **b** *NBR1* may function as a regulatory hub for plant - virus interactions. The NBR1 protein controls virus infection by targeting viral effector proteins (e.g. the VSR of TuMV) and viral particles for degradation by autophagy. The virus, in turn, triggers conversion of *NBR1* mRNA into vasiRNAs with the potential to induce widespread control over *NBR1* and other gene targets. The role of *NBR1* as a virus-inducible hub for *trans*-acting siRNA production is feedback-controlled through the ability of the same locus to also produce vasiRNAs that target the virus.

In this work, we analyzed local spreading sites of TuMV infection in *B. napus* leaves to study virus-associated sRNA regulatory nodes in two oilseed rape cultivars. Using a genome-wide approach, we identified multiple virus-encoded as well as host-encoded sRNAs that were differentially expressed during infection. Degradome data support experimentally that some of the sRNAs of viral and host origin may be effective in guiding RNA cleavage events during early stages of infection (12 dpi). Here we focus on several representative examples to depict a model (Fig. 7a) whereby viral susceptibility in plants can be explained on the basis of a genome-wide, complex and interactive sRNA-guided regulatory network that functions in a bidirectional fashion to control the expression of both viral and host genomes. The scenario whereby the host uses endogenous vasiRNAs and other hsiRNAs to target the virus and the virus uses vsiRNAs to target the host is reminiscent of cross-kingdom RNA interference (RNAi). It has been reported that fungi and parasitic plants produce sRNAs to target defense-related genes in the host^10,67,68^, whereas the host plants produce hsiRNAs that target essential virulence genes of the parasite^69,70^. Our observations indicate that the plant-virus interface recapitulates this new paradigm of molecular interactions and communication between organisms.

The vasiRNAs and other hsiRNAs with potential to target the TuMV genome originate from coding genes that convert their mRNA transcripts into secondary siRNAs. Production of secondary siRNAs has emerged as a regulatory mechanism for multiple protein-coding genes, particularly of NB-LRR disease resistance genes^71^. Consistently, in our study, several hsiRNAs predicted to guide effective targeting of the TuMV genome arose from resistance *TAO1* and *RPP5* genes. This observation suggests a dual capacity for these resistance genes to provide sRNA-based immunity as well as protein-based immunity. Furthermore, our data suggest that a broad induction of vasiRNAs may represent a basic mechanism to control gene homeostasis during infection, thus contributing to virus-host compatibility. This is illustrated by *AGO2*, *CABBP1*, and *NBR1* genes, which produce vasiRNAs upon infection likely to regulate the levels of their transcripts. AGO2 is regarded as a surrogate for AGO1 during RNA-mediated antiviral defense against some viruses. AGO2 is activated when miR403-mediated suppression of *AGO2* is inhibited by viral suppressors^72^. Interestingly, *AGO2* transcripts were induced in infected oilseed rape plants despite cleavage remained at the miR403 complementary site. This finding has two important implications. First, the HC-Pro suppressor encoded by TuMV has no inhibitory effects on AGO1-dependent miR403-guided cleavage of *AGO2* transcripts, confirming the absence of VSR activity in cells undergoing early stages of infection at the virus front. Second, given that enhanced accumulation of *AGO2* transcript in TuMV-infected plants was not due to miR403 suppression, it may account for a transcriptional activation in response to the infection. We propose that miR403 in conjunction with other *AGO2*-derived vasiRNAs contributes to maintain AGO2 under functional levels when it is transcriptionally activated in response to virus infection. This regulatory scenario recalls the post-transcriptional control of many other pathogen-responsive genes and serves to avoiding potential deleterious effects resulting from protein overexpression^2,56,57,73^. *NBR1* genes illustrate how *trans*-acting vasiRNAs are competent to target even other unrelated, secondary mRNA targets in TuMV-infected cells. Strikingly, the *B. napus* *NBR1* genes are homologs of the selective autophagy receptor protein AT4G24690/*NBR1* gene in Arabidopsis (81–84% identity at protein level). The NBR1 protein is implicated in the degradation of viral proteins and particles by autophagy and was also shown to particularly target the HC-Pro suppressor of TuMV^74^. Thus, the invading virus may usurp sRNA functions to gain widespread control over *NBR1* genes, and extensively over other genes with antiviral functions in the plant (Fig. 7b).

The production of vasiRNAs from the coding region of genes has been previously noted in Arabidopsis plants systemically infected with several unrelated viruses^2,28^. vasiRNA biogenesis is DCL4- and RNA-DIRECTED RNA POLYMERASE 1-dependent and it is suppressed by the VSR of *Cucumber mosaic virus*, but not by the VSR of TuMV. Although the vasiRNAs in *B. napus* described here may represent vasiRNAs as those reported in Arabidopsis, the genetic requirements for their production remains to be determined. Moreover, the vasiRNAs described here also include siRNAs with the potential to target mRNAs from unrelated loci. As observed for vasiRNAs in TuMV-infected Arabidopsis plants^28^, the vasiRNAs in TuMV-infected *B. napus* plants were produced irrespective of the VSR encoded by the virus. Nevertheless, although the HC-Pro VSR of potyviruses does not interfere with the production of sRNAs, it blocks their activity by sequestration and inhibition of their methylation by HUA ENHANCER 1^75^. Unlike cells in systemically infected plants, the newly infected cells within the spreading infection sites likely contain only low VSR protein levels, which presumably render a negligible impact on sRNA-guided regulation. Consistently, the activity of miR403 and of several other miRNAs in specific mRNA target cleavage could be detected (Supplementary Data 4 and 5, and Fig. 3e).

In our study, a vsiRNA similar to miR172 was identified. Both miR172 and the miR172-like vsiRNA start with an A, suggesting that they are likely recruited into the same AGO-containing effector complexes to guide silencing of the same set of targets^76–78^. miR172 is known to interact with AGO4 to direct cleavage of AP2 targets, suggesting that miR172-like vsiRNA may work alike^79^. We found that miR172 and transcript levels of the miR172-target *RAP2–7* (BnaC03g26480D and BnaC04g15640D) decreased in infected plants (Supplementary Data 5 and Supplementary Fig. 13d). This observation suggests that both miR172 and miR172-like vsiRNA may cooperate in regulating miR172 targets when miR172 levels are reduced during the infection^80^. Although there is a mismatched loop within the 5′ seed region of the miR172-like vsiRNA, several papers have reported that perfect central complementarity is not critical for strong cleavage^81,82^. Furthermore, miR172 guides sequence-specific degradation of GFP sensors carrying partially complementary sequences, including two central mismatches^83^. These findings support the idea that this vsiRNA may direct sequence-specific cleavage of miR172 targets under suboptimal complementarity. The potential of a vsiRNA to mimic miR172 is reminiscent of the miRNA mimics known to be encoded by certain animal viruses^84^.

The pattern of viral and host sRNAs and their activities in target cleavage turned out to be similar in both cultivars. However, RNA cleavage events on viral and host RNAs that occurred in a cultivar-specific manner may contribute to the different susceptibility of Tanto and Drakkar for TuMV infection. Antiviral resistance in Tanto also correlated with the induced expression of genes involved in innate immunity through PTI (e.g., genes coding for  GH3.12/PBS3 and CRK36) and SAR (e.g., genes coding for DIR1), and in the regulation of PD (e.g., genes coding for PDCB3 and CML41) (Fig. 1). These components may participate in signaling pathways of PD regulation in response to pathogens^85–87^, perhaps by inhibiting systemic TuMV movement in Tanto. Genes that are commonly downregulated in Drakkar and Tanto include a gene for DOX1 involved in the defense against aphids, and genes encoding proteins that are functionally redundant to DOX1 such as ADF3 were targeted by *NBR1*-derived siRNAs. These observations suggest that TuMV has means to overcome the expression of specific genes with potential to inhibit its propagation between plants. Our observations suggest that these genes, and also the *TAO1* and *RPP5* genes that produce virus-targeting siRNAs, as well as the *NBR1* genes that spawn virus-induced secondary siRNAs for widespread virus-induced gene regulation, are at the core of plant-TuMV interactions in oilseed rape and may represent important targets for plant engineering. In conclusion, the observations presented here describe a global scenario by which the virus activates a network of host- and virus-encoded sRNAs that are responsible for specific virus- and host-mRNA cleavage events. As the majority of them cause little changes in target mRNA levels relative to the non-infected plants, this regulatory network likely contributes to soften the detrimental effects associated to the overexpression of virus-responsive genes. Such a regulation may have evolved to prevent hypersensitive host responses and disease, and thus to create a proper relationship to guarantee both virus and host survival. Nevertheless, the functional significance of the individual virus-regulated genes and virus- and host-encoded siRNAs remains to be further studied. As *B. napus* is recalcitrant to common agroinfection or agroinfiltration techniques, such functional tests depend on the production of homozygous knockout mutants or transgenic lines. Given the gene redundancy in this allotetraploid crop, the production of transgenic RNAi or CRISPR/CAS lines may represent the most useful approach for testing specific gene functions. Functional tests could also include specific mutations in the virus. For example, the biological significance of host-derived siRNAs that target the virus could be tested by mutations in the viral target sequence. However, as mutations in the virus may likely have deleterious effects on the virus the conclusions that can be expected from such approach may be limited.

In our study, only relatively few vsiRNAs were linked to vRNA cleavage events, albeit vsiRNAs were abundantly produced from the entire vRNA. This observation has already been noted in previous studies^88^. We speculate that vsiRNAs may have little, if any, influence on vRNA cleavage in early colonized cells at the infection front. However, hsiRNAs may be present prior to virus arrival, and thus have a chance to cooperate with vsiRNAs in the targeting of vRNA.

The identified vasiRNA-producing loci deserve further analysis. The aligned siRNA patterns indicate phasing. Therefore, the exact phasing register as well as the sRNA(s) giving rise to the originating mRNA cleavage event(s) and thereby determining the starting point(s) for DCL processing remain(s) to be elucidated. However, this may be a difficult task given that vasiRNAs may target the same genes from which they are derived. These additional cleavage events likely initiate additional rounds of vasiRNA production from different starting points, thus leading to the accumulation of vasiRNAs with overlapping phasing patterns. Future studies may also reveal whether vasiRNAs are equivalent to “transcript-derived siRNAs” (ct-siRNAs) that are produced upon inhibition of RNA decay^89^, which normally eliminates aberrant transcripts arising from RNA degradation, endo-cleavage, or end processing^90^. Conceivably, virus infection could induce a disruption or overload of RNA decay pathways, thereby leading to the accumulation of aberrant transcripts that are prone to dsRNA formation and processing into siRNAs. RNA decay pathways play a role in antiviral defense and are inhibited by TuMV in *A. thaliana*^91^. Thus, it remains to be determined if vasiRNAs are produced through viral suppression of RNA decay.

## Methods

### Plant materials, plant growth conditions, virus inoculation, and RNA extraction

*B. napus* cvs Drakkar and Tanto were grown for 4–5 weeks in a growth chamber at 24 °C with a 16 h photoperiod. Drakkar leaves were rub-inoculated using freshly crude extracts prepared from *Nicotiana benthamiana* leaves systemically infected after agroinfiltration of the infectious clone pCB-TuMV-GFP^92^. pCB-TuMV-GFP (GenBank: EF028235.1) contains a GFP-tagged cDNA copy of the TuMV-UK1 isolate, which has been verified by sequencing before use. Sap extracted from Drakkar plants was then used as the source of inoculum for further experiments. Sap from Drakkar plants treated with extracts from non-inoculated *N*. *benthamiana* was used for controls (mock).

Local sites of TuMV-GFP infection in the inoculated *B. napus* leaves were monitored with a hand-held UV lamp (UVP Blak-Ray B-100) and leaf disks carrying individual infection sites were carefully isolated at 12 dpi with a 0.5 cm micropunch and immediately frozen in liquid nitrogen. Leaf disks of mock-treated Drakkar (sample DM) and mock-treated Tanto plants (sample TM), as well as of virus-treated Drakkar (sample DT) and virus-treated Tanto plants (sample TT) were dissected from three independent inoculation experiments. Thus, three biological replicates were prepared for each treatment (DM, DT, TM, and TT).

Total RNA was extracted from 200 dissected leaf disks (2 g) each using the RNeasy Mini Kit (Qiagen). The quality of each RNA sample was verified by gel electrophoresis and BioAnalyzer (Agilent technologies) techniques. The relative number of TuMV copies was determined by RT-qPCR using total RNA (30 ng) and forward (5′-TGTTCGGCTTGGATGGAA-3′) and reverse (5′-TTAACGTCCTCGGTCGTATGC-3′) primers. Each of the 12 RNA samples was used for mRNA, sRNA, and PAREseq (mRNA degradome sequencing). sRNAseq and mRNAseq sequencing libraries were constructed and sequenced (Illumina HiSeq2000) by aScidea Computational Biology Solutions (http://www.ascidea.com/). mRNA and sRNA reads were created by (2 × 100 bp) paired-end sequencing and (1 × 50 bp) sequencing, respectively.

### Degradome sequencing

PAREseq libraries were constructed by purifying mRNA from each total RNA sample (75–150 µg) using the Dynabeads mRNA direct purification kit (Ambion). Purified mRNA (1 µg) was ligated to a 5′ RNA oligonucleotide adapter (5′-GUUCAGAGUUCUACAGUCCGAC-3′) using T4 RNA ligase (New England Biolabs). The ligated products were purified using Dynabeads and used as a template for cDNA synthesis using the primer 5′-CGAGCACAGAATTAATACGACTTTTTTTTTTTTTTTTTTV-3′ and SuperScript II reverse transcriptase (Invitrogen). The resulting cDNA was amplified by PCR with primers 5′-GTTCAGAGTTCTACAGTCCGAC-3′ and 5′-CGAGCACAGAATTAATACGACT-3′, and Phusion DNA polymerase (Finnzymes) using 5 cycles of 98 °C for 20 s, 60 °C for 20 s, and 72 °C for 3 min. PCR products were gel purified by polyacrylamide gel electrophoresis (PAGE) and digested with *Mme*I^54^. The 20-nt DNA fragments resulting from *Mme*I digestion were gel purified and 3′-ligated to a double-stranded DNA adapter (top 5′-P-TGGAATTCTCGGGTGCCAAGG-3′, bottom 5′- CCTTGGCACCCGAGAATTCCANN-3′ using T4 DNA ligase. The ligation products were further purified by PAGE and amplified by PCR (21 cycles of 98 °C for 20 s, 60 °C for 20 s and 72 °C for 15 s) with indexed Illumina primers (Illumina primers RPI1 to RPI4) to allow multiplexing during sequencing. The final libraries consisting of amplicons of approximately 125 bp were again purified by PAGE. The suitability of our libraries for high-throughput sequencing was tested by cloning them into TOPO-TA vector and low-scale sequencing of individual clones. After quantification using Nanodrop, the degradome libraries were sequenced (1 × 50 bp) on an Illumina HiSeq2000 platform by aScidea.

### Whole genome re-sequencing

DNA isolated from fast-frozen leaf tissue (1.9 g) of Drakkar and Tanto plants was used for creation of 800 bp insert PCR-free libraries and subsequent paired-end sequencing (2 × 150 bp) of the Drakkar and Tanto genomes on an Illumina HiSeq 4000 platform (BGI Tech Solutions, Hongkong) with 15× coverage. The 123 Mio clean reads obtained for each cultivar were mapped (Supplementary Data 16) to the *B. napus* “Darmor-bzh” reference genome sequence (Genoscope v4.1)^40^ with the Burrows-Wheeler Aligner BWA-MEM algorithm (RRID:SCR_010910)^93^ version v0.7.15-r1142, using default parameters. Duplicated reads were marked with Picard-tools (RRID:SCR_006525; http://broadinstitute.github.io/picard/) version v1.140 and single-nucleotide polymorphism (SNP) calling (Supplementary Data 16) was performed with GATK HaplotypeCaller (RRID:SCR_001876)^94^, version v3.3-0. Only high-quality homozygous SNPs were kept using the filters: QD < 2.0 | | FS > 60.0 | | MQ < 40.0 | | MQRankSum < −12.5 | | ReadPosRankSum < −8.0 | | DP < 4. Representative genomes for Drakkar and Tanto cultivars were constructed by inferring these cultivar-specific high confidence SNPs into the reference sequence with GATK FastaAlternateReferenceMaker (RRID:SCR_001876; https://software.broadinstitute.org/gatk/documentation/tooldocs/3.8-0/org_broadinstitute_gatk_tools_walkers_fasta_FastaAlternateReferenceMaker.php). The reconstructed Drakkar and Tanto genomes were used for the rest of the analysis. In comparison to the Darmor-bzh reference genome, the Drakkar and Tanto genomes show 1.5 SNP variants per kb (Supplementary Data 16) with the large majority of variants mapping to intragenic regions (Supplementary Fig. 16). Genes were re-annotated with blastx against the *A. thaliana* genome (with default parameters) and the *Arabidopsis* gene identifiers were used to receive the GO annotations from the protein knowledgebase (UniProtKB).

### Bioinformatic analysis of mRNA, sRNA, and degradome profiles

The mRNAseq, sRNAseq, and PAREseq raw reads libraries were quality checked with FastQC (RRID:SCR_014583) version v0.11.2 and cleaned with cutadapt (RRID:SCR_011841) version v1.8.1 for adapter removal, N removal, and quality filtering. For mRNAseq reads, only those longer than 60 bp and with a Phred score >30 were kept. The clean mRNA reads (Supplementary Data 17) were mapped to the specific Drakkar and Tanto transcriptomes using Tophat2 (https://ccb.jhu.edu/software/tophat/index.shtml) version v2.0.13^95^, allowing one mismatch and applying Bowtie2 (http://bowtie-bio.sourceforge.net/bowtie2/index.shtml)^96^ as its read-alignment engine. For differential expression analysis the mapped reads were counted using the SAMtools (RRID:SCR_002105)^97^ idxstats tool version v1.1, and analyzed using Bioconductor - DESeq2 (RRID:SCR_015687)^98^ version v1.10.0. The results were filtered for expressed transcripts with at least 150 mean reads and showing statistically significant changes (*p*-value < 0.05) in expression between conditions. We assigned GO terms to our set of differentially expressed genes and used WEGO (Web Gene Ontology Annotation Plot, version 2.0)^99^ for annotation and comparison of GO results between Drakkar and Tanto using the *B. napus* GO annotation reference database (version 5.0) publicly available at the Genoscope Website (http://www.genoscope.cns.fr/brassicanapus/data/GO/). *χ*^2^-tests (*p*-values < 0.05) were done to determine which GO categories were statistically over- or under-represented in each set of differentially expressed genes that were either induced or repressed by TuMV in each cultivar.

For sRNAseq read libraries (Supplementary Data 17), clean reads that were 18–30 nts in length and which had a Phred score higher than 30 were kept and then annotated by mapping them with no mismatches to TuMV-GFP and to miRNAs of *Brassicacea* (*B. napus*, *B. oleracea*, *B. rapa*, *A. thaliana*, *A. lyrate*) compiled from miRbase^100^ using Bowtie2^96^ version v2.2.9 and applying the option “-t -N 0–end-to-end–very-sensitive–score-min C,0,0”. The set of miRNAs derived from miRbase was complemented with miRNAs described in recent publications^101–103^ and with miRNAs derived from MIR genes predicted by ShortStack (RRID:SCR_010834)^104^. We used ShortStack version v3.4. in de novo mode and the list of predicted miRNAs was filtered to keep only clusters with: Dicer call 21, 22, 24 nts, and miRNAs step Y_N15. The total list of known miRNAs contained 1047 miRNA sequences, against which the sRNA reads were aligned. sRNA reads that were strongly induced by infection and could be mapped in alignment to both strands of *B. napus* coding genes but not to the viral genome were identified as vasiRNAs (virus-activated siRNAs,^28^). We focused our analysis on vasiRNAs that were 21 nts in length and represented by at least 100 reads in the samples from infected tissues. Moreover, vasiRNA-producing genes had a PHAS Score higher than 30 (determined by ShortStack) and the normalized number of siRNAs (reads per million, r.p.m.), which could be aligned to these genes was at least twofold higher in the virus-treated sample as compared to the mock-treated control. Alignment of vasiRNAs to their gene of origin was assessed by visualization using the Integrated Genome Viewer tool IGV 2.3^105^.

The clean PAREseq libraries (Supplementary Data 17) were analyzed with PAREsnip^55^ (implemented in the UEA sRNA Workbench; http://srna-workbench.cmp.uea.ac.uk/^106^, version v4.2.1 alphaD), with min read abundance set to 100 and default parameters. sRNAseq and mRNAseq datasets were used as inputs to predict sRNA : mRNA pairs associated with identified monophosphorylated mRNA 5′ends consistent with sRNA-mediated RNA target cleavage. Specific cleavage events were normalized and compared between samples in r.p.m. (number of specific cleavage events multiplied by 10^6^ and divided by the total number of cleavage events in the degradome library). The sums of normalized cleavage events occurring in the three replicates per condition (specific cultivar/specific treatment) were used to compare specific cleavage events between conditions. The data include sRNA-associated cleavage events found in only one degradome analysis, as indicated. Readers should be aware that some of these may be false positives^55^. The gene or viral origins of the sRNAs associated with specific mRNA or vRNA cleavage events were identified by extracting the sRNA associated with a specific cleavage event from PAREsnip and by aligning its sequence to the cultivar/viral transcriptome without mismatch, using Bowtie2. A role of a given sRNA (e.g., miRNA, vasiRNA, vsiRNA) in specific RNA target cleavage was determined by searching the sRNA : RNA target pair outputs of PAREsnip with the specific sRNA query sequence(s).

### Quantitative RT-PCR

Approximately 150 leaf disk samples were pooled from ten plants per treatment and replicate, and used for total RNA extraction with Trizol reagent (Invitrogen, USA) following the manufacturer’s instructions. RNA quantities were determined by spectrophotometric analysis using Nanodrop equipment (Thermo Scientific, USA). RNA preparations (1–2 µg) were reverse transcribed using an oligo dT primer and SuperScript IV reverse transcriptase (200 U/µl) (Invitrogen). The abundance of specific transcript was measured by probing the cDNA by quantitative real-time PCR using a SYBR-green master mix (Roche) and allele-specific primers (Supplementary Data 18) in a Lightcycler 480 (Roche). Because the mRNAseq data refer to changes in the expression of specific A or C genome-specific alleles, the analysis was performed with genes for which allele-specific, intron-spanning primer sets showing no homology to any other paralogs or homologous sequences could be designed. The specific transcript levels were measured in five independent biological replicates for each cultivar in mock-treated or virus-infected conditions. Each RNA sample was analyzed by three technical replicates and specific RNA levels were normalized against the levels of transcripts produced by two known housekeeping genes [Bra011516 encoding a TIP41-like protein and BnaA08g11790D (“BnaUP1”; unknown function)]^107,108^]. Significant differences in mRNA abundance between samples were determined by an F-test (test for homoscedasticity) followed by a t-test, assuming a normal distribution of the data.

### 5′-Rapid amplification of cDNA ends

Το analyze mRNA 5′-ends by 5′-RACE^109,110^, poly(A)^+^ mRNA was purified from total RNA (1–5 µg) using a magnetic isolation module (NEBNext^®^, BioLabs) and ligated to the GeneRacer RNA oligo 5′-RACE adapter (Invitrogen) using T4 RNA ligase (New England BioLabs). The ligation product was purified using Illustra Microspin S-300 Sephacryl columns (GE Healthcare) and reverse transcribed into cDNA using an oligo(dT)_20_ primer and SuperScript IV reverse transcriptase (200 U/l, Invitrogen). Each cDNA was then used of PCR amplification using the GeneRacer 5′-RACE outer primer mapping to a sequence within the 5′-adapter and two nested, gene-specific primers (Supplementary Data 19). The PCR products were purified with a PCR clean-up kit (Macherey-Nagel) and sequenced with an ABI3130xl sequencer (Applied Life Technologies).

### Statistics and reproducibility

Data are based on biological samples derived from three independent replicate experiments. In each experiment sets of 10–12 *B. napus* cv Drakkar and 10–12 *B. napus* cv Tanto plants were infected with TuMV-GFP and similar number of plants of each cultivar were mock-treated at the same time. Roughly 200 small leaf disks were harvested and pooled for each condition and experimental replicate. For samples from infected (treated) plants, each leaf disk carried a local viral infection, thus each replicate sample provides averaged information from 200 individual infection events. RNA from each of the 12 samples [the three replicate experiments provide three independent samples of each Drakkar-mock, DM; Drakkar-treated, DT; Tanto-mock, TM; Tanto-treated (TT)] was analyzed by RNAseq, sRNAseq and PAREseq, thus resulting data are sample-specific and directly related to each other. Statistical data in relation to differential expression of mRNAs and miRNAs (e.g. mean read counts, *p*-values) are based on the combined analysis of the three replicates per condition and calculated by DEseq2 software as explained in the software manual. The significance of target-specific cleavage events has been interpreted based on the given number of degradome samples in which the specific event was found. Target-specific cleavage events found in only one of the 12 samples can be false positives. RT-qPCR experiments were performed with samples from five independent biological replicates per condition and each sample was analyzed three times (three technical replicates).

### Reporting summary

Further information on research design is available in the Nature Research Reporting Summary linked to this article.

## Supplementary information

Supplementary Information

Description of Additional Supplementary Files

Supplementary Data 1

Supplementary Data 2

Supplementary Data 3

Supplementary Data 4

Supplementary Data 5

Supplementary Data 6

Supplementary Data 7

Supplementary Data 8

Supplementary Data 9

Supplementary Data 10

Supplementary Data 11

Supplementary Data 12

Supplementary Data 13

Supplementary Data 14

Supplementary Data 15

Supplementary Data 16

Supplementary Data 17

Supplementary Data 18

Supplementary Data 19

Reporting Summary

## Data Availability

Data used to support the conclusions are presented in the figures and the Supplementary Information. Original mRNAseq, sRNAseq, and PAREseq data are available at the NCBI Sequence Read Archive (SRA) under accession code PRJNA508739.
