# Supplementary material for: Turnip mosaic virus in oilseed rape activates networks of sRNA-mediated interactions between viral and host genomes
Source: Commun Biol. 2020 Nov 23;3:702. doi: 10.1038/s42003-020-01425-y (PMC7683744; doi:10.1038/s42003-020-01425-y)
Supplement: Supplementary file 1 — Supplementary Information [file 42003_2020_1425_MOESM1_ESM.pdf]

Supplementary Figures

Turnip mosaic virus in oilseed rape activates networks of sRNA-mediated interactions between viral and host genomes

Nicolas Pitzalis, Khalid Amari, Stéfanie Graindorge, David Pflieger, Livia Donaire, Michael Wassenegger, César Llave, and Manfred Heinlein

Supplementary Figure 1: GO annotation enrichment analysis of down-regulated genes

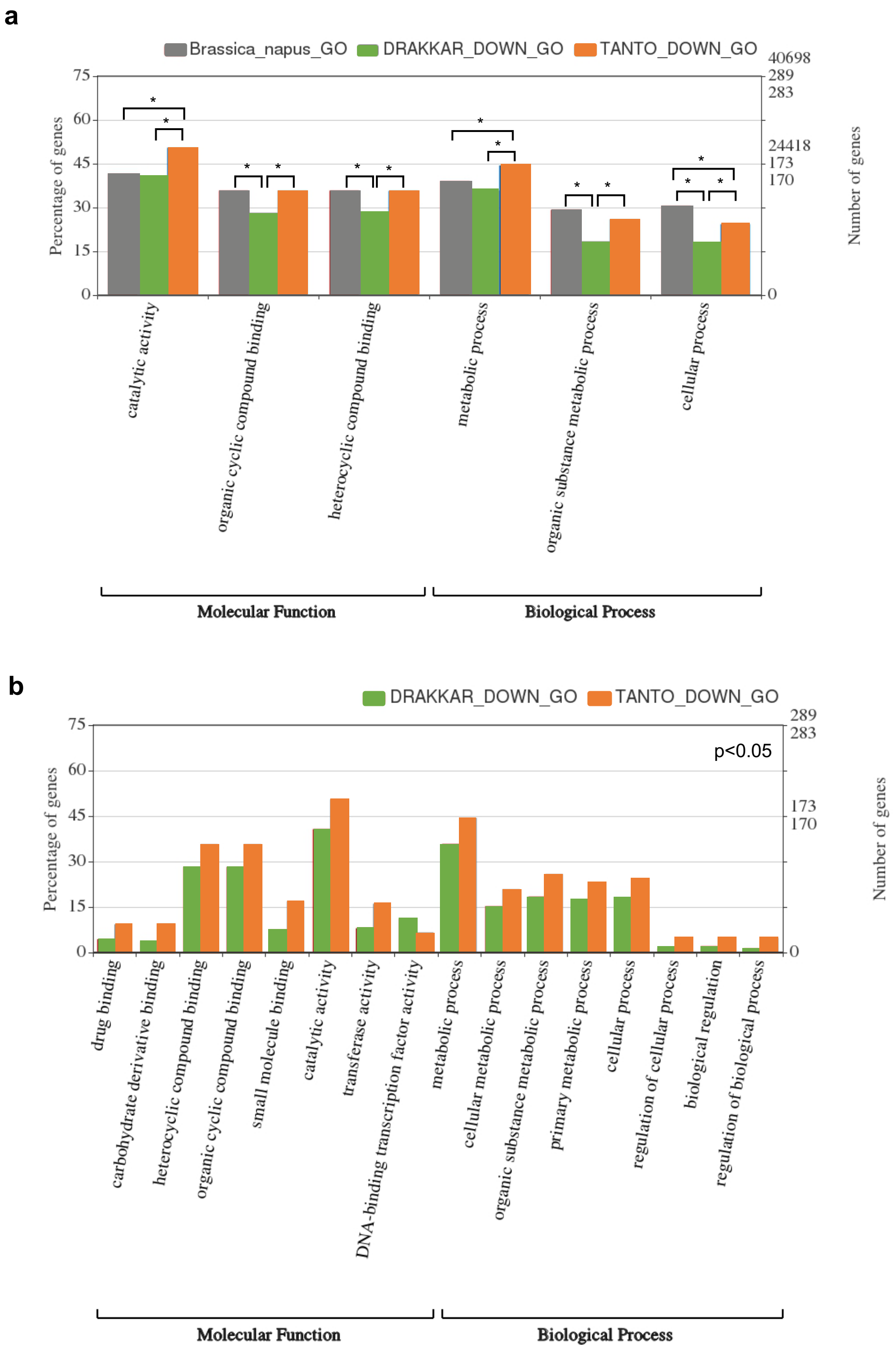

Gene ontology (GO) enrichment analysis. WEGO distributions of GO terms with genes that were down-regulated in Drakkar and Tanto cultivars after TuMV infection. The plots show percent and number of overrepresented GO terms under cellular component, molecular function and biological process categories. **a** GO terms with the most significant differences compared to the reference transcriptome (*Brassica napus*). Asterisks indicate the statistically significant (Chi-square test, $p < 0.05$) enrichment of important GO categories. **b** GO terms with the most significant differences between Drakkar and Tanto (Chi-square test, $p < 0.05$).

Supplementary Figure 2: GO annotation enrichment analysis of up-regulated genes

a

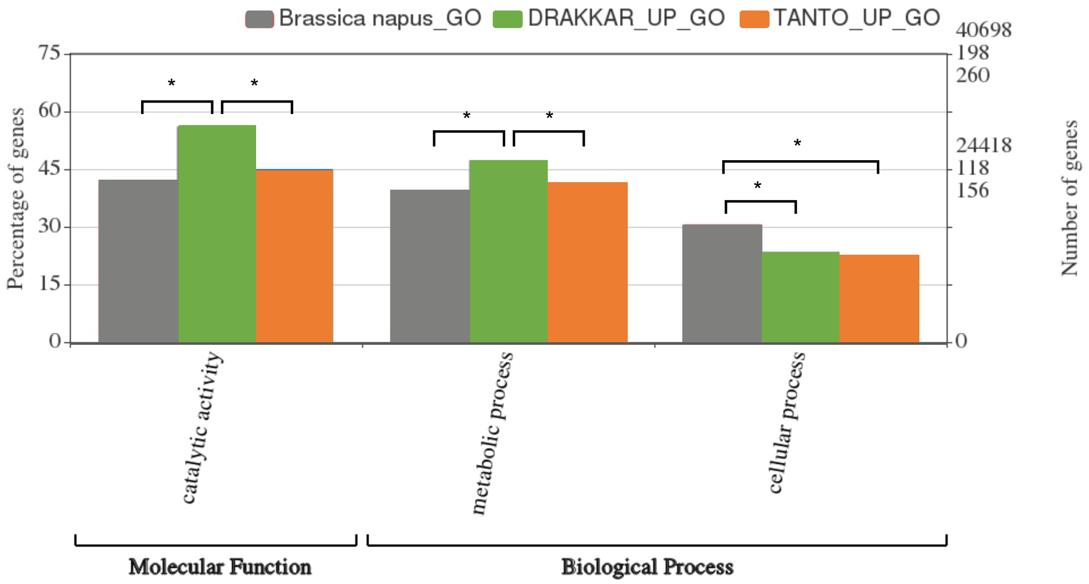

b

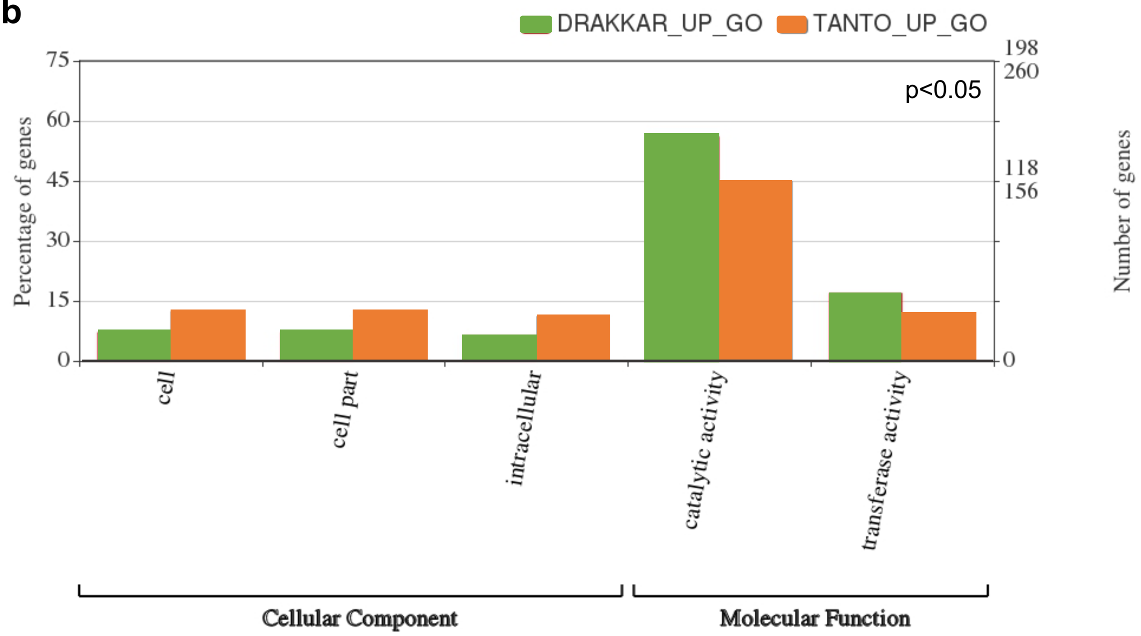

Gene ontology (GO) enrichment analysis. WEGO distributions of GO terms with genes that were up-regulated in Drakkar and Tanto cultivars after TuMV infection. The plots show percent and number of overrepresented GO terms under cellular component, molecular function and biological process categories. **a** GO terms with the most significant differences compared to the reference transcriptome (*Brassica napus*). Asterisks indicate the statistically significant (Chi-square test, $p < 0.05$) enrichment of important GO categories. **b** GO terms with the most significant differences between Drakkar and Tanto (Chi-square test, $p < 0.05$).

Supplementary Figure 3: Confirmation of RNAseq-based gene expression data by RT-qPCR

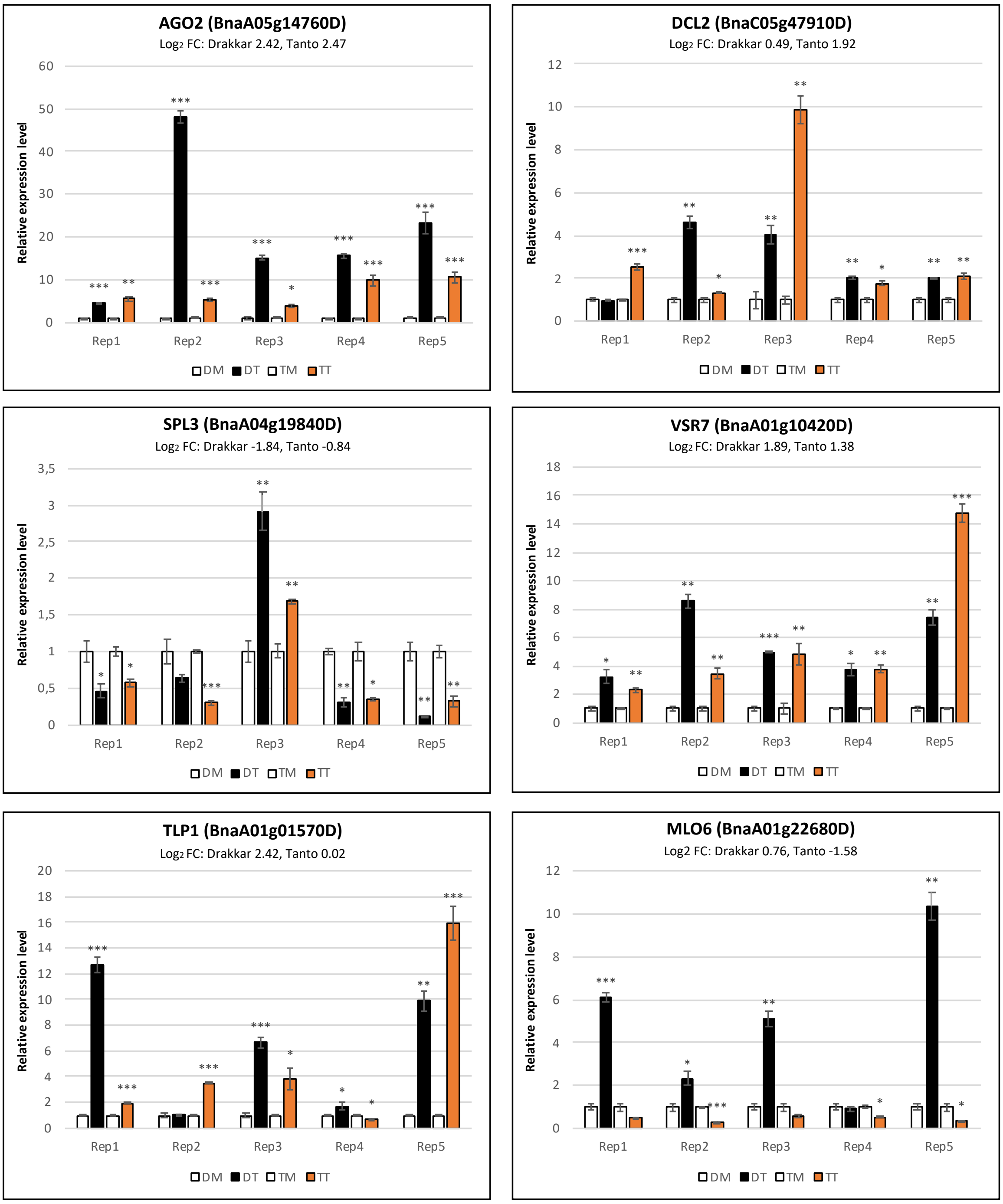

Confirmation of RNAseq-based gene expression data by RT-qPCR. Relative expression levels in local sites of virus-infected leaves (DT, TT) compared to mock-control tissues (DM, TM; set to 1) are shown. RT-qPCR assays were performed with original polyA RNA used for RNAseq (Rep1) as well as with polyA RNA isolated from new biological samples derived from four new independent experimental replicates (Rep2-Rep5). Error bars represent the standard error between technical replicates (N= 3). The statistical significance levels between control (mock, white columns) and treated (virus infected) samples (black and brown columns) are indicated for each experimental replicate by asterisks: $p<0.05$ = *; $p<0.01$=**; $p<0.001$ = ***.

Supplementary Figure 4: Uncropped, original image of agarose gel

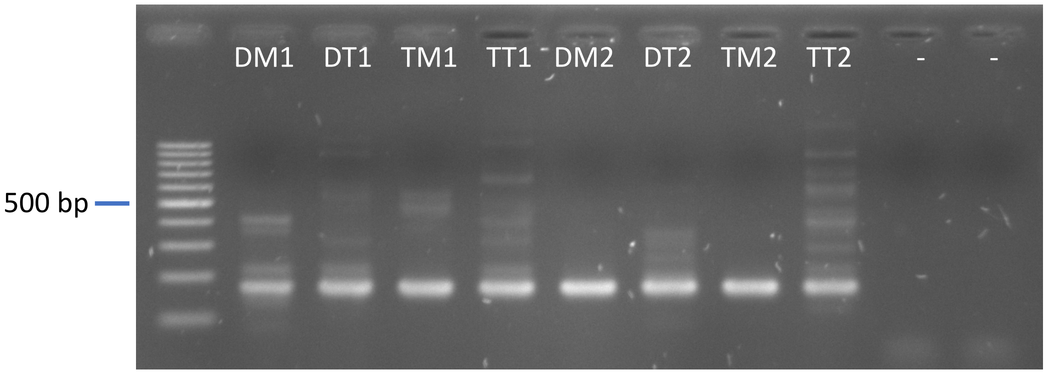

Uncropped original of the agarose gel image shown in Figure 3e. The GeneRuler 100 bp DNA Ladder (ThermoFisher) was used as size marker.

Supplementary Figure 5: Sequence-specific cleavage of *ARF10* mRNA

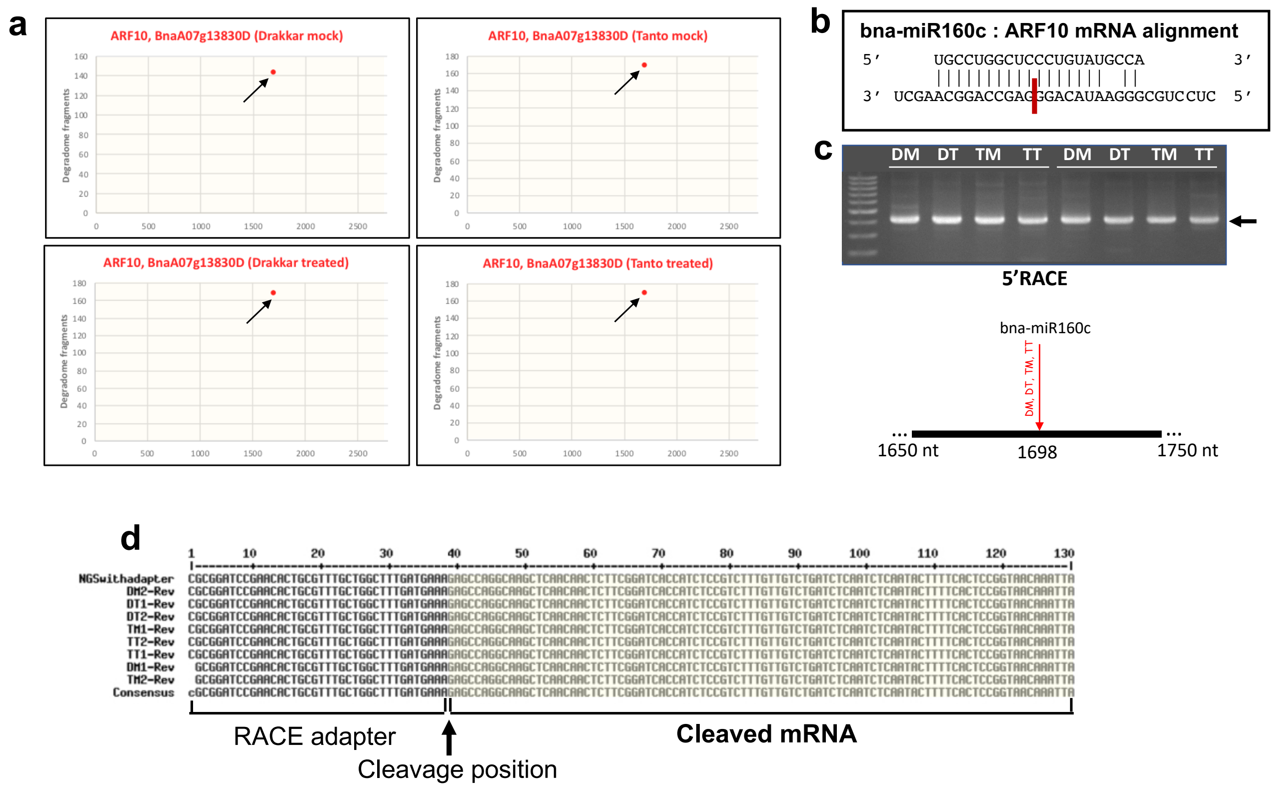

Sequence-specific cleavage of *ARF10* mRNA at the precited bna-miR160c-binding site. **a** Target plots showing 5' signature abundance along the ARF10 mRNA (BnaA07g13810D) identified through degradome sequencing of mock-treated and infected Drakkar and Tanto. Arrows in the plots indicate the site of bna-miR160 binding at position 1698. Y-axis shows the total number of 5' degradome signatures from three replicate samples per condition. X-axis represents the length of the *ARF10* mRNA. **b** Base-pair alignment between bna-miR160c and *ARF10* mRNA. The preferential cleavage site at nucleotide 1698 is indicated by a red line. **c** 5' RACE analysis of independent biological samples confirmed the exact cleavage site at position 1698 of the transcript. The electrophoresis gel shows a 5' RACE amplified product of the expected size (arrow). **d** Verification of the cleavage site by cDNA sequencing of the 5' RACE amplification fragment. DM, mock-inoculated Drakkar; DT, TuMV-infected Drakkar; TM, mock-inoculated Tanto; TT, TuMV-infected Tanto.

Supplementary Figure 6: Uncropped, original image of agarose gel

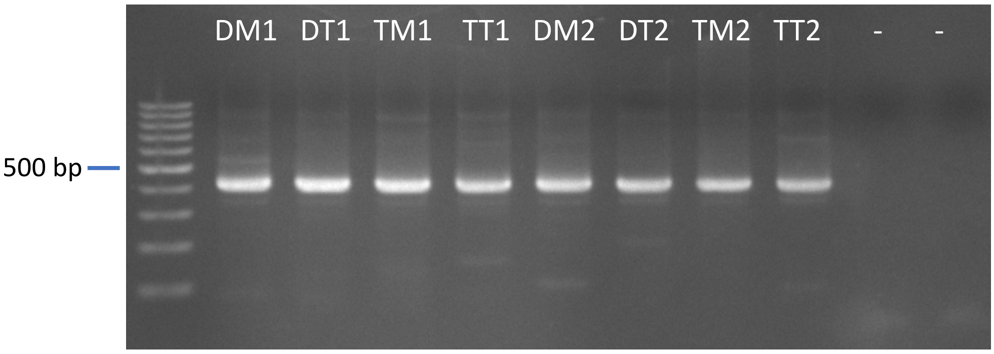

Uncropped original of the agarose gel image shown in Supplementary Figure 5c. The GeneRuler 100 bp DNA Ladder (ThermoFisher) was used as size marker.

Supplementary Figure 7: Sequence-specific cleavage of *APETALA2* (AP2) mRNA

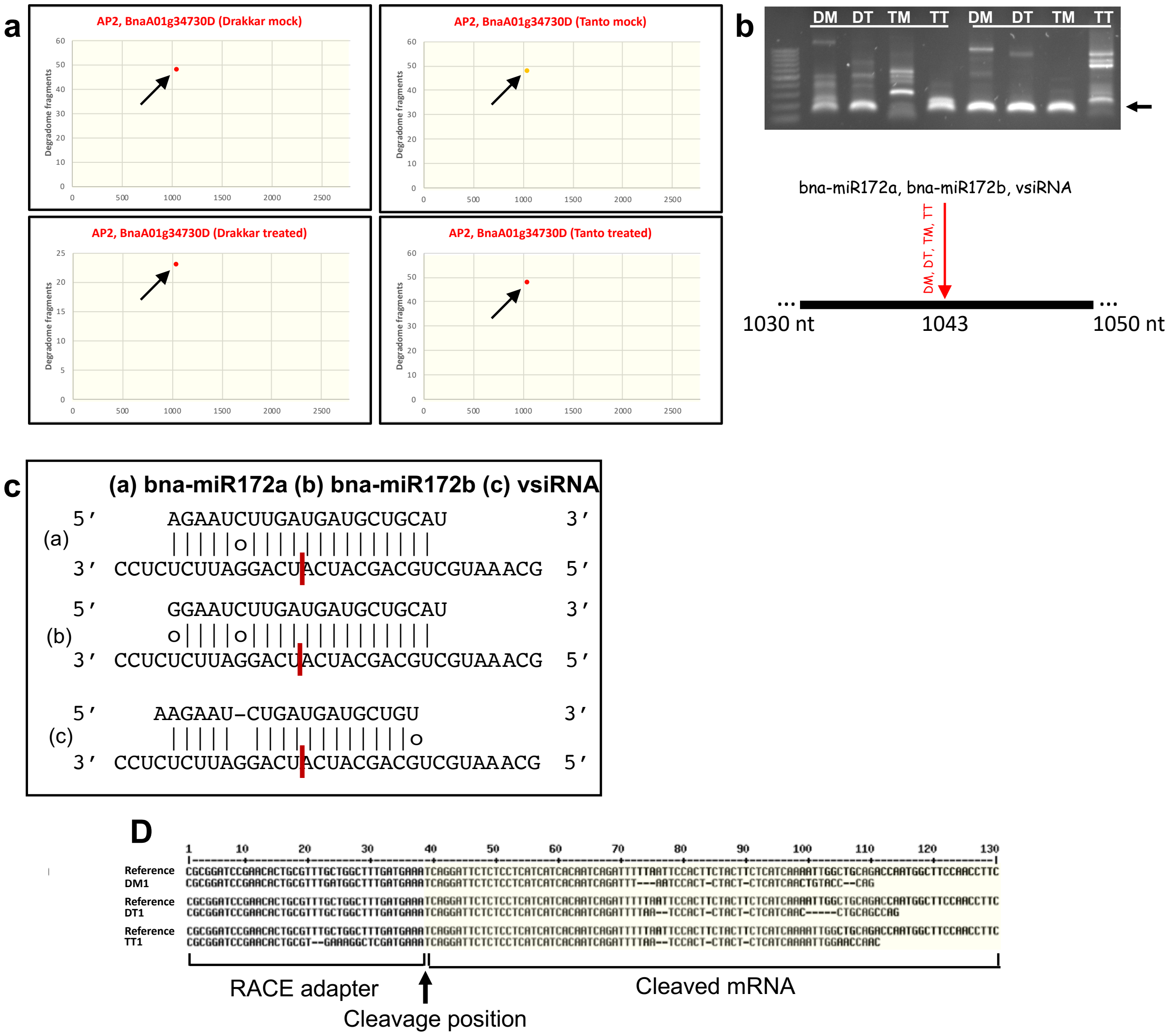

Sequence-specific cleavage of *APETALA2* (AP2) mRNA. **a**Target plots showing 5' signature abundance along the ARF10 mRNA (BnaA07g13810D) identified through degradome sequencing of mock-treated and infected Drakkar and Tanto. Arrows in the plots indicate a preferential processing site at position 1043 consistent with cleavage by bna-miR172a, bna-miR172b or TuMV vsiRNA. Y-axis shows the total number of 5' degradome signatures from three replicate samples per condition. X-axis represents the length of the *AP2* mRNA mRNA. **b** Base-pair alignment between *AP2* mRNA and bna-miR1472a (a), bna-miR172b (b) and a specific TuMV vsiRNA (c). The specific cleavage at nucleotide positions 1043 is indicated by a red line. **c** 5' RACE analysis of independent biological samples confirmed the exact cleavage sites at this mRNA position. The electrophoresis gel shows a major 5' RACE amplification product of the expected size (arrow). **d** The specific cleavage site at position 1043 of the mRNA was confirmed by cDNA sequencing of the 5' RACE amplification fragment. DM, mock-inoculated Drakkar; DT, TuMV-infected Drakkar; TM, mock-inoculated Tanto; TT, TuMV-infected Tanto.

Supplementary Figure 8: Uncropped, original image of agarose gel

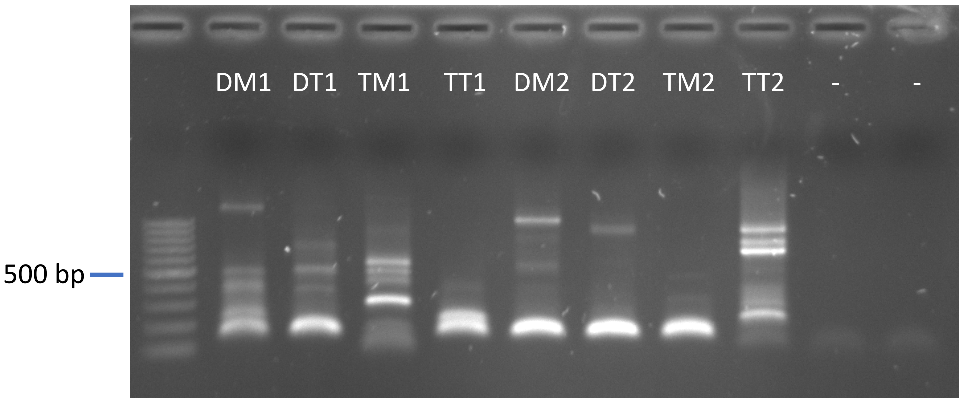

Uncropped original of the agarose gel image shown in Supplementary Figure 7b. The GeneRuler 100 bp DNA Ladder (ThermoFisher) was used as size marker.

Supplementary Figure 9: Sequence-specific cleavage of *BL1S2* mRNA

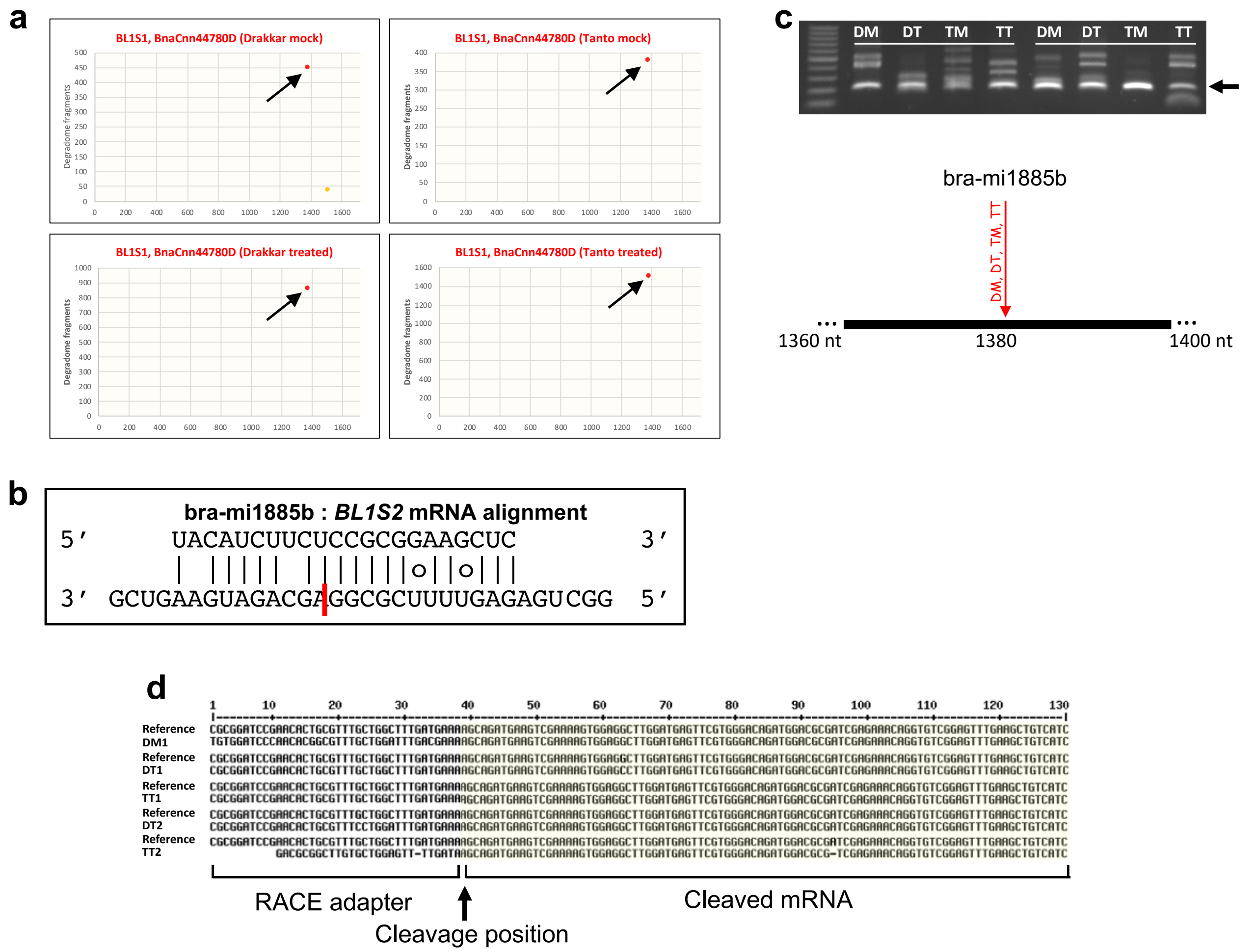

Sequence-specific cleavage of *Biogenesis of Lysosome-related organelles complex 1 subunit 2 (BL1S2)* mRNA at the predicted bra-miR1885b-binding site. **a** Target plots showing 5' signature abundance along the *BL1S2* mRNA (BnaCnng44780D). identified through degradome sequencing of mock-treated and infected Drakkar and Tanto. Arrows in the plots indicate the site of bra-miR1885b binding at position 1380. Y-axis shows the total number of 5' degradome signatures from three replicate samples per condition. X-axis represents the length of the *BL1S2* mRNA mRNA. **b** Base-pair alignment between bra-miR1885b and *BL1S2* mRNA. The specific cleavage site at nucleotide positions 1380 is indicated by a red line. **c** 5' RACE analysis of independent biological samples confirmed the precise cleavage sites at this position. The electrophoresis gel shows a major 5' RACE amplification product of the expected size (arrow). **d** The specific cleavage site at position 1380 of the mRNA was confirmed by cDNA sequencing of the 5' RACE amplification fragment. DM, mock-inoculated Drakkar; DT, TuMV-infected Drakkar; TM, mock-inoculated Tanto; TT, TuMV-infected Tanto.

Supplementary Figure 10: Uncropped, original image of agarose gel

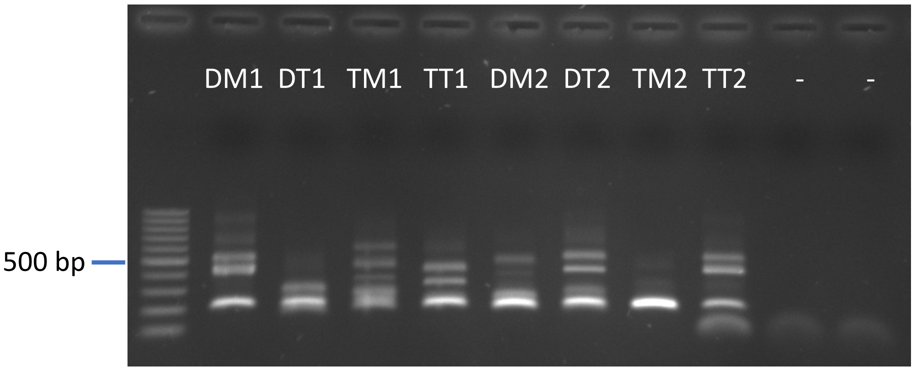

Uncropped original of the agarose gel image shown in Supplementary Figure 9c. The GeneRuler 100 bp DNA Ladder (ThermoFisher) was used as size marker.

Supplementary Figure 11: Sequence-specific cleavage of *Target of Avtb Operation 1* (TAO1) mRNA

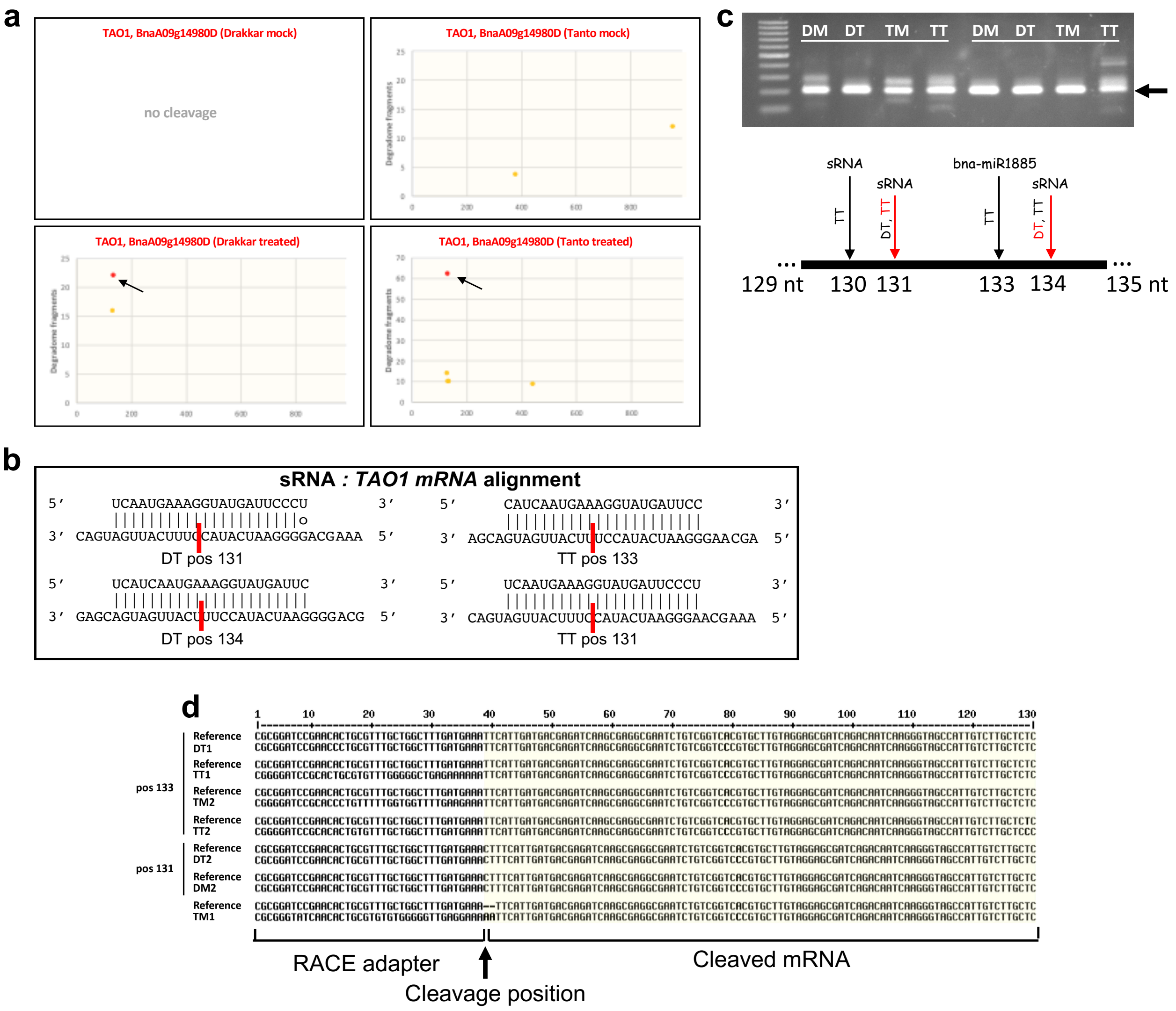

Sequence-specific cleavage of *Target of Avtb Operation 1 (TAO1)* mRNA. **a** Target plots showing 5' signature abundance along the *TAO1* mRNA (BnaA09g14980D) identified through degradome sequencing of mock-treated and infected Drakkar and Tanto. Arrows in the plots indicate preferential processing sites at positions 131-134 consistent with cleavage by bna-miR1885a and related sRNAs. Y-axis shows the total number of 5' degradome signatures from three replicate samples per condition. X-axis represents the length of the *TAO1* mRNA. **b** Base-pair alignment between *TAO1* mRNA and bna-miR1885a. The specific cleavage sites at nucleotide positions 131-134 are indicated by a red line. **c** 5' RACE analysis of independent biological samples confirmed the exact cleavage sites at these positions of the transcript. The electrophoresis gel shows a major 5' RACE amplification product of the expected size (arrow). **d** The specific cleavage sites were confirmed by cDNA sequencing of the 5' RACE amplification fragments. DM, mock-inoculated Drakkar; DT, TuMV-infected Drakkar; TM, mock-inoculated Tanto; TT, TuMV-infected Tanto.

Supplementary Figure 12: Uncropped, original image of agarose gel

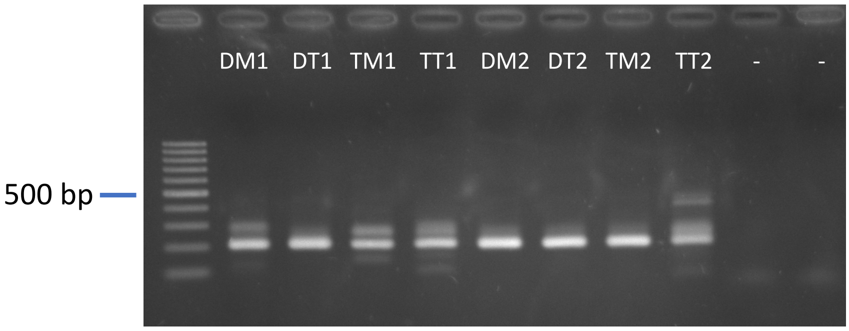

Uncropped original of the agarose gel image shown in Supplementary Figure 11c. The GeneRuler 100 bp DNA Ladder (ThermoFisher) was used as size marker.

Supplementary Figure 13: Sequence-specific cleavage of *RAP2-7* mRNA

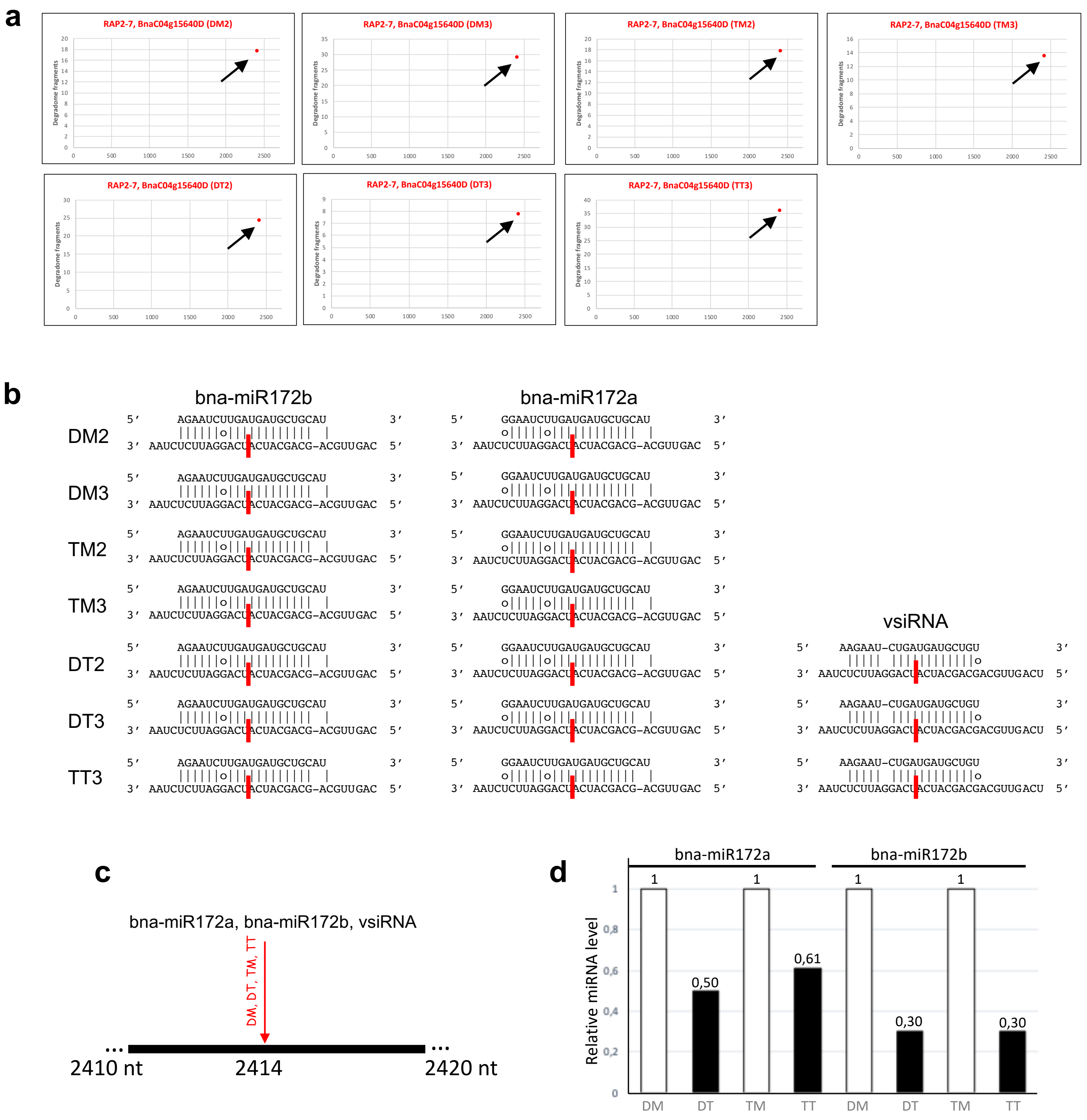

Sequence-specific cleavage of *RAP2-7* mRNA. **a** Target plots showing 5' signature abundance along the *RAP2-7* mRNA (BnaC04g15640D) identified through degradome sequencing of mock-treated and infected Drakkar and Tanto. Arrows in the plots indicate preferential processing sites at position 2414 consistent with cleavage by bna-miR172a*,* bna-miR172b, and TuMV vsiRNA. Y-axis shows the total number of 5' degradome signatures in single replicate samples per condition as indicated. X-axis represents the length of the *RAP2-7* mRNA. **b** Base-pair alignment between *RAP2-7* mRNA and bna-miR172a, bna-miR172b and the vsiRNA as identified in samples reported in A. The specific cleavage site at position 2414 is indicated by a red line. **c** Location of the specific cleavage site at nucleotide positions 2414. **d** Mean relative accumulation levels of bna-miR172a and bna-miR172b in TuMV-infected relative to mock-inoculated (set at 1) Drakkar and Tanto plants as determined by sRNAseq. DM, mock-inoculated Drakkar; DT, TuMV-infected Drakkar; TM, mock-inoculated Tanto; TT, TuMV-infected Tanto.

Supplementary Figure 14: vasiRNA-producing genes

BnaA05g14760D (AGO2)

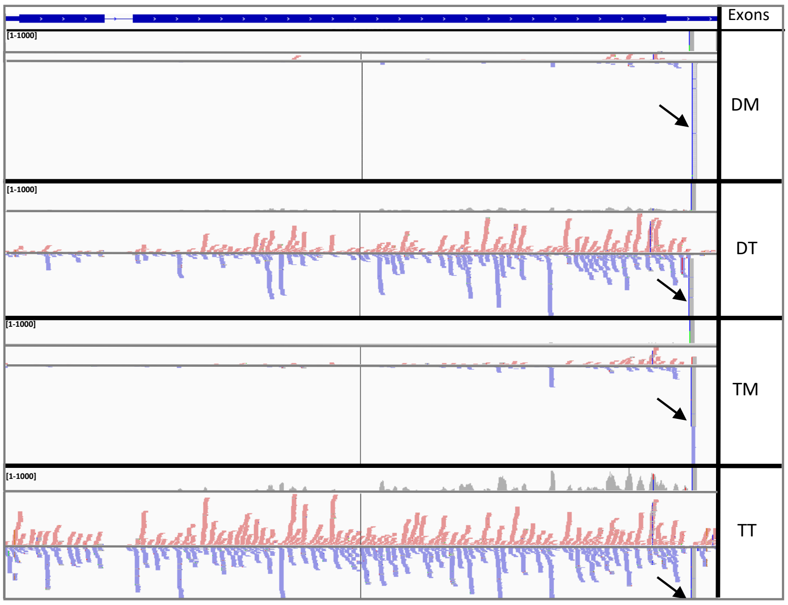

BnaA05g28710D (CDC48E)

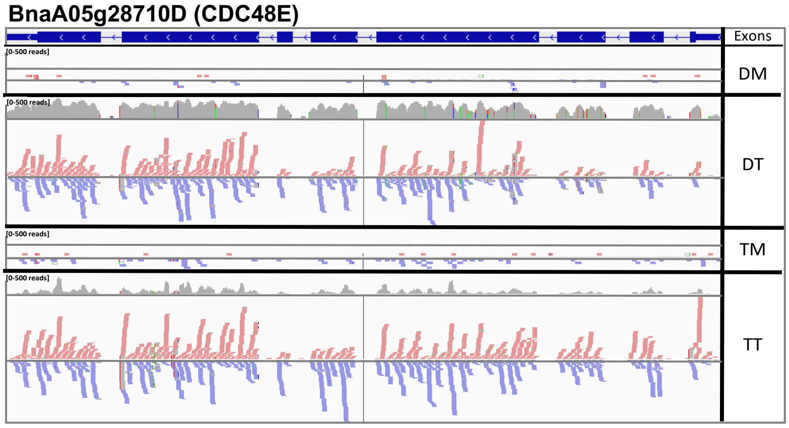

BnaC02g40260D (EF2, Elongation factor 2)

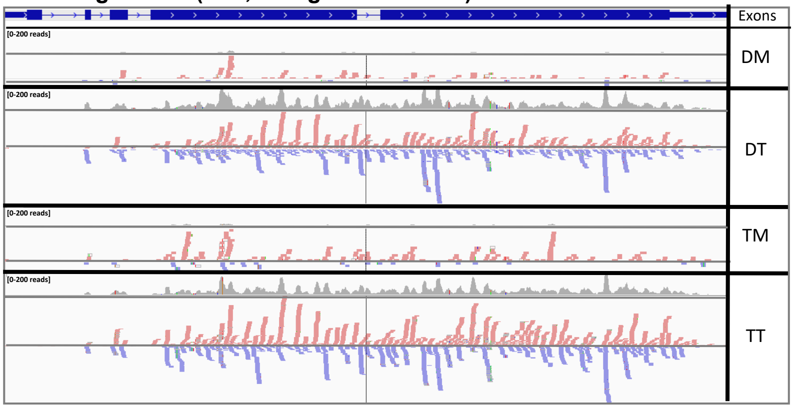

Examples of vasiRNA-producing genes. The exon-intron structure of several representative vasiRNA-generating transcripts (AGO2, CDC48E and EF2) and mapping of unique sRNAs sequenced in TuMV-infected and mock-inoculated Drakkar and Tanto samples are shown. Unique sRNA reads derived from the positive and negative strand of the dsRNA are depicted in red and blue, respectively. The distribution of total sRNA reads is shown in grey. Arrows indicate miR403 reads. DM, mock-inoculated Drakkar; DT, TuMV-infected Drakkar; TM, mock-inoculated Tanto; TT, TuMV-infected Tanto.

Supplementary Figure 15: Representative loci producing trans-acting siRNAs associated with vRNA cleavage events

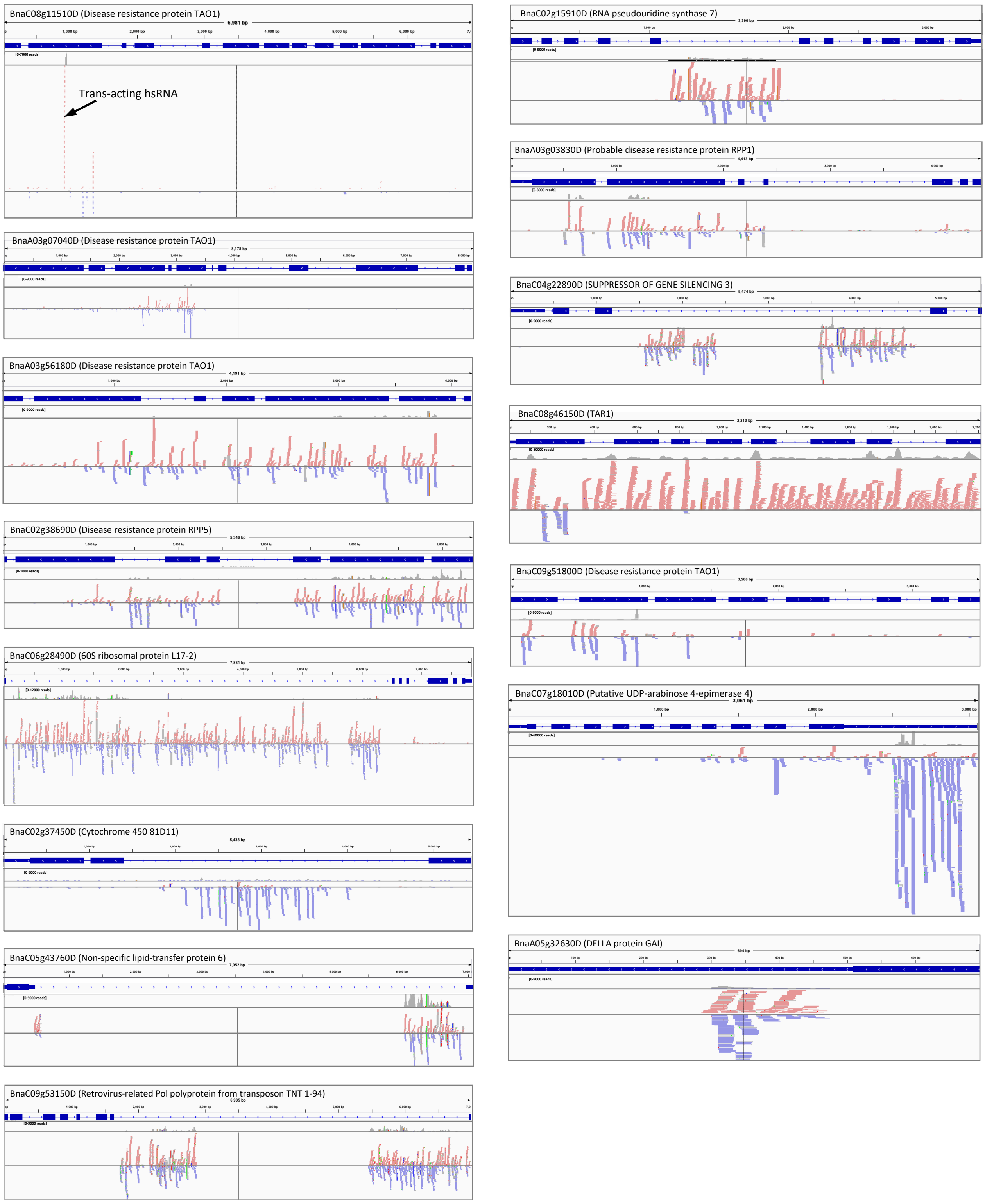

Representative loci producing trans-acting siRNAs associated with viral RNA cleavage events. The different panels show the identity of the host siRNA (hsiRNA)-producing gene, the exon-intron structure of the mRNA and the alignment of hsiRNAs to the mRNA. The panels show hsiRNAs from mock-treated Drakkar samples. Thus, unlike vasiRNAs, the hsiRNAs shown here are expressed also in the absence of infection.

Supplementary Figure 16: Homozygous SNPs by which *B. napus* cvs. Drakkar and Tanto differ form the reference sequence of *B. napus* cv. Darmor-bzh

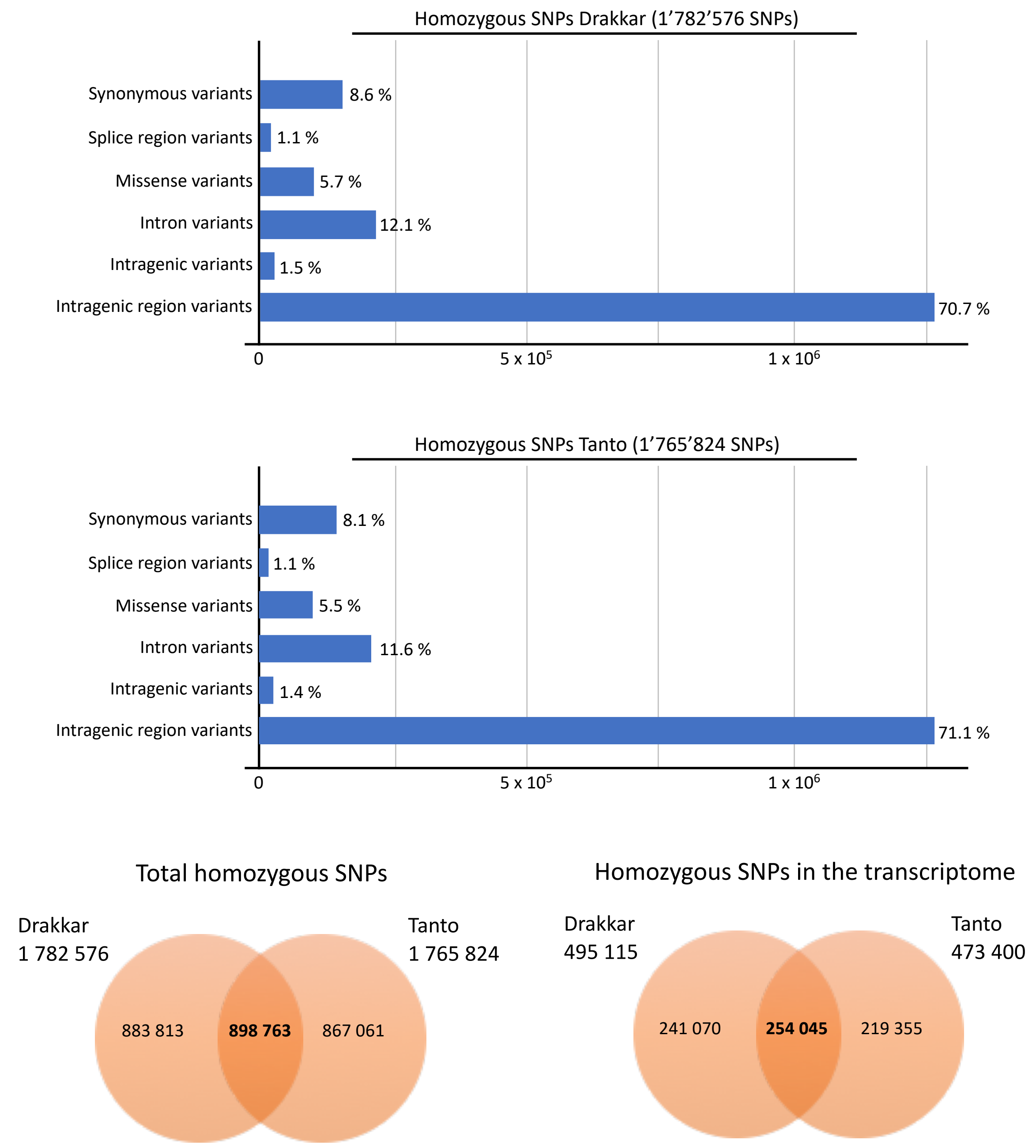

Homozygous SNPs by which B. napus cvs. Drakkar and Tanto differ form the reference sequence of *B. napus* cv. Darmor-bzh.
