# Supplementary material for: Turnip mosaic virus in oilseed rape activates networks of sRNA-mediated interactions between viral and host genomes
Source: Commun Biol. 2020 Nov 23;3:702. doi: 10.1038/s42003-020-01425-y (PMC7683744; doi:10.1038/s42003-020-01425-y)
Supplement: Supplementary file 2 — Description of Additional Supplementary Files [file 42003_2020_1425_MOESM2_ESM.pdf]

Description of Additional Supplementary Files

File Name: Supplementary Data 1
Description: mRNA transcripts that were most strongly and significantly regulated within sites of local TuMV infection in Drakkar and Tanto leaves. These genes responded significantly to TuMV infection in both Drakkar and Tanto. The fold-change (FC) values (log2) represent significant differences between transcript levels within infection sites and transcript levels in mock-treated tissue samples (p-value: <0.05). These values are based on three independent infection experiments (biological replicates) for both Drakkar and Tanto and the standard error for the estimated log2 FC (lfcSE) is shown. In each experiment, transcripts were detected with at least 150 mean reads.

File Name: Supplementary Data 2
Description: mRNA transcripts showing most strongly and significant cultivar-specific regulation within sites of local TuMV infection in leaves. The fold-change values (log2) represent significant differences between transcript levels within infection sites and transcript levels in mock-treated tissue samples (p-value: <0.05). These values are based on three independent infection experiments (biological replicates) for both Drakkar and Tanto and the standard error for the estimated log2 FC (lfcSE) is shown. In each experiment, transcripts were detected with at least 150 mean reads. Only genes with strongest fold-changes ([log2] +4 / -4) are shown.

File Name: Supplementary Data 3
Description: Changes in miRNA expression levels of TuMV infection in Drakkar and Tanto. The fold-change values (log2) represent significant differences between miRNA levels within infection sites and miRNA levels in mock-treated tissue samples (p-value: <0.05). These values are based on three independent infection experiments (biological replicates) for both Drakkar and Tanto and the standard error for the estimated log2 FC (lfcSE) is shown. In each experiment, unique miRNAs were detected with at least 10 mean reads.

File Name: Supplementary Data 4
Description: miRNA-mediated mRNA target cleavage found in at least three independent PAREseq analysis samples.

File Name: Supplementary Data 5
Description: miRNA-mediated mRNA target cleavage associated with significant fold-changes (p <0,05) in miRNA and mRNA target levels upon infection in Drakkar and Tanto.

File Name: Supplementary Data 6
Description: Targeting of host mRNA transcripts by vsiRNAs (vsiRNA:mRNA target pairs). Indexes refer to the indexed vsiRNAs in Figure 2b. vsiRNA:mRNA target pairs found in only one PAREseq replicate may be false positives.

File Name: Supplementary Data 7
Description: Targeting of host mRNA transcripts by vsiRNAs. vsiRNAs associated with mRNA target cleavage found in several PARE replicates. Indexes refer to the indexed vsiRNAs in

Figure 2b. A specific vsiRNA (g) that targets several ERF genes in Drakkar and Tanto is highlighted in bold. vsiRNA:mRNA target pairs are shown Supplementary Data 6.

File Name: Supplementary Data 8
Description: vasiRNA-producing loci in Drakkar and Tanto ranked by number of vasiRNA reads.

File Name: Supplementary Data 9
Description: vasiRNA-producing loci common to Drakkar and Tanto and ranked by number of vasiRNA reads.

File Name: Supplementary Data 10
Description: vasiRNA production from AGO2, CABBP1 and NBR1 loci.

File Name: Supplementary Data 11
Description: vasiRNAs derived from CABBP1 and NBR1 genes and associated with mRNA target cleavage.

File Name: Supplementary Data 12
Description: mRNA targets of vasiRNAs derived from CABBLP1 and NBR1 genes.

File Name: Supplementary Data 13
Description: Examples of trans-acting vasiRNAs.

File Name: Supplementary Data 14
Description: TuMV vsiRNAs associated with viral RNA cleavage in Drakkar and Tanto

File Name: Supplementary Data 15
Description: hsiRNAs associated with viral RNA cleavage in Drakkar and Tanto.

File Name: Supplementary Data 16
Description: DNA sequencing statistics and variant calling

File Name: Supplementary Data 17
Description: RNAseq, sRNAseq, and PAREseq read and mapping statistics

File Name: Supplementary Data 18
Description: Oligonucleoide primers used for RT-qPCR analysis.

File Name: Supplementary Data 19
Description: Oligonucleotide adapters and primers used for 5' RACE analysis.
