# Supplementary material for: Turnip mosaic virus in oilseed rape activates networks of sRNA-mediated interactions between viral and host genomes
Source: Commun Biol. 2020 Nov 23;3:702. doi: 10.1038/s42003-020-01425-y (PMC7683744; doi:10.1038/s42003-020-01425-y)
Supplement: Supplementary file 22 — Reporting Summary [file 42003_2020_1425_MOESM22_ESM.pdf]

nature research

Corresponding author(s): Manfred HEINLEIN, César LLAVE

Last updated by author(s): Sep 25, 2020

# Reporting Summary

Nature Research wishes to improve the reproducibility of the work that we publish. This form provides structure for consistency and transparency in reporting. For further information on Nature Research policies, see our Editorial Policies and the Editorial Policy Checklist.

## Statistics

For all statistical analyses, confirm that the following items are present in the figure legend, table legend, main text, or Methods section.

| n/a | Confirmed | |
|---|---|---|
| ☐ | ☒ | The exact sample size ($n$) for each experimental group/condition, given as a discrete number and unit of measurement |
| ☐ | ☒ | A statement on whether measurements were taken from distinct samples or whether the same sample was measured repeatedly |
| ☐ | ☒ | The statistical test(s) used AND whether they are one- or two-sided<br>*Only common tests should be described solely by name; describe more complex techniques in the Methods section.* |
| ☒ | ☐ | A description of all covariates tested |
| ☒ | ☐ | A description of any assumptions or corrections, such as tests of normality and adjustment for multiple comparisons |
| ☐ | ☒ | A full description of the statistical parameters including central tendency (e.g. means) or other basic estimates (e.g. regression coefficient) AND variation (e.g. standard deviation) or associated estimates of uncertainty (e.g. confidence intervals) |
| ☐ | ☒ | For null hypothesis testing, the test statistic (e.g. $F$, $t$, $r$) with confidence intervals, effect sizes, degrees of freedom and $P$ value noted<br>*Give P values as exact values whenever suitable.* |
| ☒ | ☐ | For Bayesian analysis, information on the choice of priors and Markov chain Monte Carlo settings |
| ☒ | ☐ | For hierarchical and complex designs, identification of the appropriate level for tests and full reporting of outcomes |
| ☒ | ☐ | Estimates of effect sizes (e.g. Cohen's $d$, Pearson's $r$), indicating how they were calculated |

*Our web collection on statistics for biologists contains articles on many of the points above.*

## Software and code

Policy information about availability of computer code

Data collection: Original sequencing data were obtained by Illumina sequencing. Data provided by the seqeuncing company were raw sequencing data.

Data analysis: All software used and listed below is described in the Methods section of the manuscript.

Burrows-Wheeler Aligner BWA-MEM algorithm (RRID:SCR_010910) version v0.7.15-r1142
Picard-tools (RRID:SCR_006525; http://broadinstitute.github.io/picard/) version v1.140
GATK HaplotypeCaller (RRID:SCR_001876) version v3.3-0
GATK FastaAlternateReferenceMaker (RRID:SCR_001876; https://software.broadinstitute.org/gatk/documentation/tooldocs/3.8-0/org_broadinstitute_gatk_tools_walkers_fasta_FastaAlternateReferenceMaker.php)
FastQC (RRID:SCR_014583) version v0.11.2
cutadapt (RRID:SCR_011841) version v1.8.1
Tophat2 (https://ccb.jhu.edu/software/tophat/index.shtml) version v2.0.13
Bowtie2 (http://bowtie-bio.sourceforge.net/bowtie2/index.shtml)
SAMtools (RRID:SCR_002105) idxstats tool version v1.1
Bioconductor - DESeq2 (RRID:SCR_015687) version v1.10.0
ShortStack RRID:SCR_010834)
PAREsnip (implemented in the UEA sRNA Workbench (http://srna-workbench.cmp.uea.ac.uk/; version v4.2.1 alphaD))

For manuscripts utilizing custom algorithms or software that are central to the research but not yet described in published literature, software must be made available to editors and reviewers. We strongly encourage code deposition in a community repository (e.g. GitHub). See the Nature Research guidelines for submitting code & software for further information.

## Data

Policy information about availability of data

All manuscripts must include a data availability statement. This statement should provide the following information, where applicable:

- Accession codes, unique identifiers, or web links for publicly available datasets
- A list of figures that have associated raw data
- A description of any restrictions on data availability

Data used to support the conclusions are presented in the figures and the supplementary information. Original mRNAseq, sRNAseq, and PAREseq data are available at the NCBI Sequence Read Archive (SRA) (https://www.ncbi.nlm.nih.gov/sra/PRJNA508739). All other relevant source data, such as DEseq2, PAREsnip, and ShortStack output data can be retrieved from a data depository (DOI:10.5281/zenodo.4043257).

# Field-specific reporting

Please select the one below that is the best fit for your research. If you are not sure, read the appropriate sections before making your selection.

☒ Life sciences ☐ Behavioural & social sciences ☐ Ecological, evolutionary & environmental sciences

For a reference copy of the document with all sections, see nature.com/documents/nr-reporting-summary-flat.pdf

# Life sciences study design

All studies must disclose on these points even when the disclosure is negative.

| | |
|---|---|
| Sample size | The data described in this manuscript are based on plant RNA samples derived from four experimental conditions (DM, DT, TM, TT). For each condition three independent experimental replicates were analyzed (12 samples in total). Each of the 12 samples contains RNA from 200 individually leaf disks harvested from ten same-treated plants. Each of the 12 samples was subdivided into three samples for analysis by sRNAseq, RNAseq, and PAREseq. The RNAseq and sRNAseq results of the three experimental replicates per experimental condition were statistically analyzed and compared by DEseq2 software for differential RNA and sRNA expression between experimental conditions (standard errors and p-values are provided). The RNAseq, sRNAseq and PAREseq results of each replicate and experimental condition were analyzed by PAREsnip software for the identification of cleavage sites in RNA, thus resulting in 12 PAREseq replicates. The number of PAREseq replicates in which a specific cleavage event was found is provided in the tables. RNA samples for quantitative RT-PCR were derived from independent experiments and each sample contained RNA from 150 leaf disks. DNA of 1.9 g fast-frozen leaf tissue of each cultivar was used for DNA re-sequencing. |
| Data exclusions | No data are excluded. However, Tables show data selected based on parameters described in the text and Legends. |
| Replication | RNAseq, sRNAseq, and PAREseq experiments were performed three times, each with fully independent biological material. Degradome data are based on 12 independent PAREseq libraries (3x DM; 3x DT; 3x TM; 3x TT). For some genes, RNAseq data ( based on three replicate experiments) were confirmed by quantitative RT-PCR assays four times each with fully independent biological material. 5'RACE results showing miRNA-directed cleavage of specific mRNAs are confirmed by the PAREseq/PAREsnip data (the PAREseq/PAREsnip data are themselves based on the analysis of replicative samples; see 'sample size'). |
| Randomization | Four different experimental conditions were addressed (DM, DT, TM, TT) with samples of independent replicative experiments for each condition. Samples belonging to the same condition were equally treated to generate means values, standard errors and p-values. Results obtained for each condition were compared to each other to generate differential expression data. |
| Blinding | During sampling, the 150-200 individual leaf samples (leaf disks carrying individual sites of virus infection, and leaf control samples of the same size) collected for each RNA sample were equal in appearance in the respective category (infected, non-infected) and randomly selected. There was no means to influence the outcome of the experiment. Thus, no blinding was applied. |

# Reporting for specific materials, systems and methods

We require information from authors about some types of materials, experimental systems and methods used in many studies. Here, indicate whether each material, system or method listed is relevant to your study. If you are not sure if a list item applies to your research, read the appropriate section before selecting a response.

## Materials & experimental systems

| n/a | Involved in the study |
| --- | --- |
| ☒ | ☐ Antibodies |
| ☒ | ☐ Eukaryotic cell lines |
| ☒ | ☐ Palaeontology and archaeology |
| ☒ | ☐ Animals and other organisms |
| ☒ | ☐ Human research participants |
| ☒ | ☐ Clinical data |
| ☒ | ☐ Dual use research of concern |

## Methods

| n/a | Involved in the study |
| --- | --- |
| ☒ | ☐ ChIP-seq |
| ☒ | ☐ Flow cytometry |
| ☒ | ☐ MRI-based neuroimaging |
